# Degradation Kinetics for Organic Nitrogen in Bioelectrochemical Systems toward Ammonia Recovery

**DOI:** 10.1021/acsestengg.6c00128

**Published:** 2026-03-31

**Authors:** McKenzie Burns, Ziyan Wu, Tia Mirsha, Andrew Beaudet, Katie Mangus, Mohan Qin

**Affiliations:** Department of Civil and Environmental Engineering, 5228 University of Wisconsin−Madison, Madison, Wisconsin 53706, United States

**Keywords:** bioelectrochemical systems, ammonia recovery, organic nitrogen mineralization, degradation kinetics

## Abstract

Land application of dairy manure returns nitrogen (N) to the soil for crop production. However, direct land application of manure faces challenges such as nitrogen volatilization and imprecise manure nitrogen applications, which significantly contribute to losses to the environment while also reducing the nitrogen value of manure. Manure processing methods that can recover nitrogen, particularly organic nitrogen (orgN) in a mineralized form, in a concentrated product can increase the nutrient use efficiency, reducing the demand for manufactured nitrogen fertilizers. In this study, we investigate two operation configurations of bioelectrochemical systems (BES) for ammonia (NH_3_) recovery from orgN in synthetic dairy manure. Glutamic acid, an amino acid found in high concentrations in dairy manure, was used as the N source in the synthetic feed, and the BES was operated in both microbial electrolysis cell (MEC, *E*_appl._ = 0.8 V) and microbial fuel cell (MFC) operation modes. Samples from four time series experiments, two in each operation mode, were analyzed for chemical oxygen demand (COD), total nitrogen (TN), total ammoniacal nitrogen (TAN), and acetate concentrations. Raman spectroscopy was applied to track the orgN content in the time series samples throughout the experiments. Results indicated superior N removal from the anolyte in MEC mode, with an average TN removal above 95% and first-order degradation kinetics with rate coefficients between 0.05 and 0.06 h^–1^. Kinetic analysis of the Raman data revealed that glutamic acid degradation to be complex and not singularly ordered in either operation mode, requiring further quantitative study. This work provides vital insight into the kinetics of degradation within BES toward a more complete understanding of anode-chamber processes. Such insight can be useful in guiding further research into BES as resource recovery mechanisms and supporting BES adaptation as manure treatment processes focused on the recovery of nutrient-rich, value-added fertilizer products.

## Graphical abstract

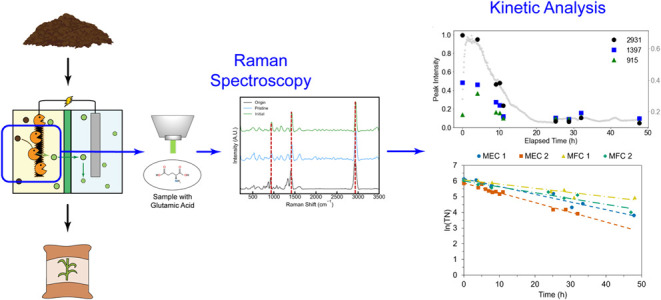

## 1 Introduction

In response to the anticipated global population increase to nearly 10.3 billion by 2085, dairy herds are expanding with the total number of active dairy cows expected to surpass 9.5 million in 2025.^1,2^ Increasing pressure on agricultural systems to provide food for the growing population has led to a heightened dependence on nitrogen-based fertilizers. The agriculture sector, comprising both livestock and cropland operations, is responsible for about 15% of global greenhouse gas emissions, with nitrogen (N) fertilizers contributing roughly 3% of that total through both their production and on-farm application emissions.^3,4^ The growing dairy industry presents a unique opportunity for nitrogen recovery, as animal wastes contain high concentrations of nitrogen, which, if recovered, could reduce demand for manufactured fertilizers.

A great portion of the nitrogen in dairy manure exists in the organic form (orgN), such as proteins, amino acids, and other complex molecules from undigested feed and microbial biomass.^5–7^ Applying dairy manure directly to crop fields to utilize this N fraction is challenging, as orgN is not immediately available to plants but must first be broken down by microbes through mineralization to inorganic forms, such as ammonium (NH_4_^+^). Manure nutrient recovery systems show a tremendous amount of potential for increasing nitrogen use efficiency and decreasing the negative environmental impacts of manure application. One emerging technology aimed at recovering NH_3_ from livestock manure is bioelectrochemical systems (BES), which were initially evaluated to recover NH_3_ from wastewater.^8–10^ BES implements microorganisms in electrochemical cells to facilitate the oxidation–reduction reactions needed to degrade contaminants into less hazardous and/or more useful forms.^11^

Both microbial fuel cells (MFCs) and microbial electrolysis cells (MECs), two forms of BES, have been highlighted in research to effectively recover NH_3_ from livestock and animal waste.^12–15^ In these BES configurations, organic contaminants are oxidized at the anode through microbial metabolism, generating electrons which are transferred through an external circuit to the cathode.^16^ A cation exchange membrane (CEM) separating anode and cathode compartments simultaneously permits the transport of cations from the anolyte to the catholyte, which is largely driven by the electromotive force created from the influx of electrical current to the cathode.^17^ Several studies have highlighted the effectiveness of BES as an NH_3_ recovery technology when the feedstock has abundant ammoniacal nitrogen; however, investigation into NH_3_ recovery in BES when initial nitrogen is in the organic form is limited.^10,18–21^ For BES operated for NH_3_ recovery from manure (of which most nitrogen is in the organic form), the microbial degradation of orgN at the anode produces NH_4_^+^, which is then transported to the cathode via the electromotive force. While other cations besides the target NH_4_^+^ ion are permitted to transport across the membrane due to the nonspecific nature of the CEM, previous studies have indicated that NH_4_^+^ is the dominant ion transported when it is present at higher concentrations than other competing cations, like Na^+^, K^+^, and Mg^2+^.^5^ At the cathode, a reduction reaction consumes electrons and creates a basic environment. In BES targeting NH_3_ recovery, this step allows for the conversion of NH_4_^+^ to NH_3_, which is easily further recovered through methods such as gas aeration and acid sorption. A schematic of the BES and relevant cell processes is included in Figure 1A.

**Figure 1:**
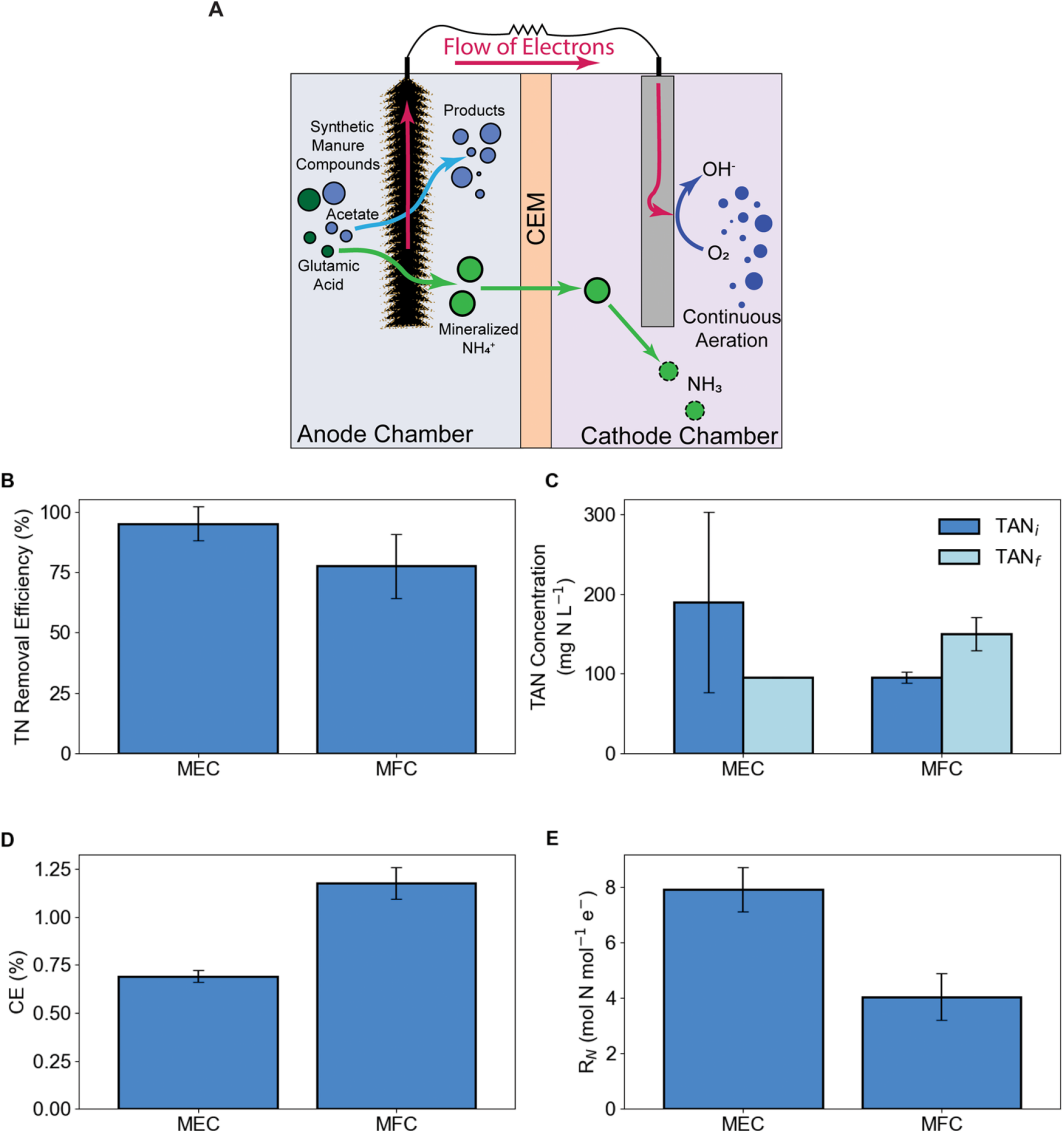
Process schematic (A) and aggregate analysis of time series experiment, including TN removal efficiency (B), initial and final anolyte total ammoniacal nitrogen (TAN) concentrations (C), Coulombic Efficiency (CE) (D), and nitrogen loading ratio (*R*_N_) (E). Bars represent averages of duplicate experiments, and error bars represent standard deviations. *X*-axis labels indicate the operation mode of the system.

Organic nitrogen, despite being highly concentrated in dairy manure, remains poorly understood within existing manure treatment technologies.^5,22,23^ This is because mineralization of orgN to inorganic forms (i.e., NH_4_^+^, NO_3_^–^) is a relatively ubiquitous microbial process.^24–26^ The field of soil science has extensively investigated orgN mineralization, particularly in relation to the carbon-to-nitrogen ratio in various soil environments and how this ratio affects microbial community composition and function.^25,27–29^ This is not typically a concern in manure treatment systems for two reasons: (1) manure contains exceedingly high concentrations of organic carbon, and (2) many manure treatment technologies require mixing and agitation, which helps to continuously disperse organics throughout the suspended microbial communities or past fixed-growth communities frequently, eliminating the concerns of transport-limited availability of organics. Furthermore, conclusions regarding orgN mineralization in the field of soil science cannot be directly adopted and assumed to apply in manure treatment systems, considering the wide variations in both soil and manure compositions, the different microbial communities present in each environment, and the sensitivity of orgN mineralizing organisms under various conditions.

Understanding orgN mineralization in BES treating manure is challenging, as there are many different forms of orgN within manure. The majority of orgN in manure is proteins and amino acids, with a significant fraction of urea from urine.^7,30,31^ The speciation of proteins and amino acids in manure is subject to variability based on several factors, including the species of cow, diet, supplements, medications, etc.^31–33^ Due to the sheer number of possible orgN compounds present in manure, aggregate measurements are often used instead of targeted analysis. Organic nitrogen concentrations are calculated by the difference between total nitrogen (TN) and total Kjeldahl nitrogen (TKN; sum of organic plus ammoniacal nitrogen).^34^ However, the wide range of variability in nitrogen content in large quantities of manure makes it challenging to consistently obtain representative samples. This variability is inconducive to kinetic studies, and as such, a more accurate and targeted measurement method is warranted. Raman spectroscopy, a nondestructive vibrational spectroscopic technique, has been widely used as a chemical probe to detect organic chemicals in water matrices due to the fast analytical speed and reliable accuracy.^35^ Compared to other traditional analytical methods deploying chromatography-based techniques to detect orgN, Raman lends itself well to targeted analysis of manure orgN since it does not require complicated sample pretreatment and can provide the analytical results within minutes.^35,36^ Here, we present a novel application of Raman spectroscopy as a noncontact optical probe to evaluate the orgN content of and investigate orgN mineralization in mixed-substrate BES samples.^37^ To aid in the simplification of the Raman measurements, we choose to use a synthetic manure substrate with a specific orgN target compound, glutamic acid, in this study. Glutamic acid is an amino acid found in high concentrations in dairy manure and is a complex, zwitterionic compound consisting of a 5-carbon chain with two carboxyl groups and one amine group.^31^

This work, which follows a previous study proving the viable application of bioelectrochemical systems for NH_3_ recovery from real dairy manure substrates,^5^ investigates the degradation of orgN in a bioelectrochemical system operated for NH_3_ recovery from synthetic dairy manure. We used a synthetic dairy manure substrate with acetate as an organic carbon source and glutamic acid as an organic carbon and nitrogen source without additional inorganic N in the feed. Time series (TS) experiments were conducted in both microbial electrolysis cell (MEC) and microbial fuel cell (MFC) operation modes, with the primary difference between operation modes being the presence of an externally applied potential difference of 0.8 V in the MEC mode. This potential difference is hypothesized to enhance NH_3_ recovery in the BES via increased transport of NH_4_^+^ from anolyte to catholyte. Furthermore, aeration of the catholyte in both operation modes is used to ensure continuous removal of TAN species (as NH_3_) from the catholyte via air stripping, further incentivizing NH_4_^+^ transport across the CEM and therefore also incentivizing continuous microbial orgN mineralization for NH_4_^+^ production in the anolyte. The objectives of this study were to (1) investigate the degradation of orgN in MFC and MEC operated for NH_3_ recovery, (2) calculate degradation constants for COD, TN, and orgN in the BES under different operation modes using Raman spectroscopy, and (3) suggest an optimal operation mode for enhanced orgN removal in the BES for dairy manure treatment. To our knowledge, this study is one of the first to investigate the kinetics of orgN mineralization in different configurations of BES, as well as to apply Raman spectroscopy as a method for detecting orgN in mixed-substrate samples.

## 2 Materials and Methods

### 2.1 Experimental Section

#### 2.1.1 BES Construction and Operation

Reactor design followed that described by Burns and Qin.^19^ To facilitate selective ion exchange, the system utilized a cation exchange membrane (CMI 7000S, Membranes International, Inc.) to separate the anode and cathode chambers. The anode is a carbon brush (The Mill-Rose Company, Mentor, OH) cultivated for microbial growth with seed from a local wastewater treatment plant (Nine Springs Wastewater Treatment Plant, Madison, WI), and the cathode is a carbon cloth coated with a platinum–carbon catalyst (surface area = 39 cm^2^). The electrodes were connected through an external 10 Ohm resistor. The electrolyte solutions are recirculated continuously in batch mode from external reservoirs at a rate of 76 mL min^–1^. The current generation was monitored continuously using a real-time data acquisition system from Keithley Instruments (Tektronix, Beaverton, OR). For experiments in electrolysis operation, an external potential difference of 0.8 V was applied between electrodes across the resistor using an external power supply (Circuit Specialists, Tempe, AZ).

Four time series (TS) experiments were run for 48 h each. Data from two TS experiments were collected under each operation condition as experimental replicates (MEC 1 and 2, MFC 1 and 2). In between TS experiments, the reactor was run in maintenance mode, where electrolyte compositions (see Section 2.1.2) and feeding schedules remained consistent with TS experiments, but no data was collected. For each TS experiment, pristine anolyte and catholyte solutions were prepared, and samples of the pristine anolyte were collected and stored for later analysis. At the start of each experiment, the anolyte and catholyte recirculation bottles (∼300 and 600 mL, respectively) were replaced, constituting a partial replacement of the entire anolyte solution volume (∼500 mL total) and a complete replacement of the catholyte solution volume. The new electrolyte was allowed to recirculate through the reactor for ∼5 min prior to initial sampling. Samples were collected initially and at hours 4, 8, 24, 28, 32, and 48 (approximately), with the sample at 48 h being the final sample in the TS experiment. To collect the samples, a syringe was connected to the reactor via tubing with a stopper. The stopper was opened, the syringe line was purged 3–5 times, and ∼10 mL of anolyte sample was collected. Samples were frozen at −80 °C between collection and analysis.

#### 2.1.2 Electrolyte Compositions

The synthetic manure anolyte was prepared in deionized (DI) water followed by the addition of 1.5 g of sodium acetate (Ward’s Science, St. Catharines, Ontario, Canada), 3.7473 g of L-(+)-glutamic acid sodium monohydrate (JT Baker, Randor, Pennsylvania), 5 mL of stock solution,^19^ 1 mL of 1 M phosphate buffer solution (Fisher Chemical, Fair Lawn, New Jersey), and 0.5 mL of trace solution.^19^ It is important to note that no inorganic nitrogen was provided in the synthetic manure solution. The prepared solution was transferred to a wide-neck media bottle and purged with N_2_ gas for 10 min immediately prior to use. The catholyte was a concentrated NaOH solution (pH 10–12, Ward’s Science, St. Catharines, Ontario, Canada) in DI water. The high pH catholyte was maintained in order to keep the catholyte free of microorganisms and also served to limit the concentration of H^+^ ions available at the cathode for use in the hydrogen evolution reaction (HER) in MEC mode, thus limiting the effects of H_2(g)_ production. The catholyte was continuously aerated through in-line aeration in both operating modes (Figure 1A). Atmospheric oxygen (roughly 21% of the O_2_) was provided continuously through an aquarium aeration pump connected directly to the catholyte influent tubing. This continuous aeration provides abundant oxygen for the oxygen reduction reaction at the cathode, minimizing limiting effects on maximum current density that may have been exhibited due to the high overpotential of the ORR. Additionally, this aeration serves as a method to continuously remove TAN (as NH_3_) from the catholyte, which aids in preventing back-diffusion of TAN species and further incentivizes transport of NH_4_^+^ from anolyte to catholyte, thus having the potential to further enhance orgN degradation.

### 2.2 Analytical

#### 2.2.1 Constituent Measurements

Chemical oxygen demand, total nitrogen, and total ammoniacal nitrogen concentrations were determined using standard colorimetric methods (COD Digestion Vials High Range, Total Nitrogen Persulfate Digestion Test ’N Tube, and High Range Ammonia Nitrogen AmVer Salicylate Test ’N Tube, Hach, Loveland, CO). For TAN measurements using this method, it is important to note that a certain amount of variability in measurements is expected due to the nature of the test, particularly compared with more precise quantification methods using advanced instrumentation. Given the basic pH and aerated conditions in the catholyte, a substantial portion of NH_3_ is expected to be stripped out and recovered, which was not quantified in this study but has been thoroughly documented elsewhere.^14,38,39^ Instead, we use the reduction in anolyte TN and TAN to represent the theoretical NH_3_ recovery. Acetate, nitrite, and nitrate concentrations in each time series sample were measured via high-performance liquid chromatography (HPLC). An AT *vp* system from Shimadzu Scientific Instruments (Columbia, MD) equipped with a Restek Ultra Aqueous C18 column (4.6 mm × 150 mm x 5 μm, Restek Corporation, Center County, PA) was used for analysis. Nitrite and nitrate concentrations in all samples from both anolyte and catholyte were consistently below 1 ppm, therefore considered negligible and not reported in this work.

#### 2.2.2 Raman Analysis

A confocal Raman spectrometer (Horiba XploRA PLUS) was used to characterize the changes in chemical components during the degradation process. Before Raman analysis, each sample was filtered through a 0.2 μm membrane to remove microbial cells, EPS, and suspended particulates. 100 μL of the filtered sample was drop-cast on a piece of aluminum foil and air-dried. Raman analysis was then performed on the dried samples. Raman spectra within the mapping area were averaged and normalized by the maximum Raman band.

#### 2.2.3 Calculations

Aggregate cell performance during each time series experiment was assessed based on changes in concentration of constituents in the anolyte solution from initial to final measurements. Removal efficiency for COD and TN was calculated according to eq 1

1 $$R=\frac{C_{i}-C_{f}}{C_{i}}\times 100$$

where *R* is the percent removal, *C*_i_ is the initial anolyte concentration of the constituent of interest, and *C*_f_ is the final anolyte concentration of the constituent of interest. Electrochemical performance was evaluated using the total charge transferred, *Q*, and the Coulombic Efficiency, CE, calculated by eqs 2 and 3, respectively

2 $$Q=\int_{0}^{t}I\times \mathrm{dt}$$

where *I* is the current (in amperes) at time *t* and

3 $$\mathrm{CE}=\frac{{\mathrm{MW}}_{O_{2}}Q}{4FV_{\mathrm{an}}\Delta \mathrm{COD}}$$

where MW_O2_ is the molecular weight of oxygen, 4 is the number of electrons exchanged per mole of COD (oxygen) consumed, *F* is Faraday’s constant (96,485 Coulombs per mole of electrons), *V*_an_ is the total anolyte volume, and ΔCOD is the change in COD concentration over the duration of the experimental cycle.^5,19^ The fraction of current production that is used to drive nitrogen transport (as NH_4_^+^) from anode to cathode chambers across the CEM is called the nitrogen loading ratio, *R*_N_, and is calculated via eq 4

4 $$R_{N}=\frac{V_{\mathrm{an}}F\left(\Delta \mathrm{TN}\right)}{Q}$$

where ΔTN is the change in TN concentration over the duration of the experimental cycle.^19^ For each parameter used to describe aggregate cell performance (*R*, CE, and *R*_N_), averages and standard deviations were calculated from experimental data obtained under the same operating mode. In reporting these statistical values, uncertainty (as standard deviation) is reported to 2 significant figures, and the average value is reported to the same number of decimal places as the uncertainty value for that measurement.

#### 2.2.4 Time Series Kinetic Analysis

Kinetic analysis was performed to investigate the kinetics of the degradation of COD, TN, acetate, and orgN within the cell. For each constituent, linear regressions were performed on unmodified and modified (natural logarithm and inverse) concentrations (or Raman band intensity, in the case of orgN measurements) versus time plots. Acceptable linearity was defined as having an *R* ^2^-value ≥ 0.85. In the case where no iterations of unmodified or modified data exhibited adequate linearity, the constituent was deemed to have complex kinetics outside the scope of this study.

## 3 Results and Discussion

### 3.1 Operation Mode Comparison

For each TS experiment, fresh synthetic manure was fed into the reactor at the start of the 48 h experimental period. Anolyte samples from throughout the experiment were analyzed for COD, TN, TAN, and acetate concentrations (see Section 2.2.1). Electrochemical performance metrics, including the nitrogen loading ratio (*R*_N_) and Coulombic efficiency (CE), were assessed as well (eqs 2, 3, and 4). Averages and standard deviations were taken across data obtained from experiments operated in the same operation mode. Values are reported in the format of average ± standard deviation.

#### 3.1.1 COD, TN, and TAN Removal

In all experiments, COD removal from the synthetic manure anolyte was excellent, ranging from an average of 98.9% ± 1.5% in MEC mode to an average of 96.2% ± 3.7% in MFC mode (see Figure S1 in the Supporting Information). This removal is reflected in the quick decreases observed in acetate concentration (a large contributor to COD) over each TS experiment, which dropped to undetectable amounts within the first 24 h of each experiment (see Figure S2 in the Supporting Information). Excellent COD removal is a common observation in BES operated with synthetic anolyte, as the simplicity of organic compounds in synthetic substrates makes it easy for microorganisms in the anode to degrade, and was also observed previously in the same system with a different synthetic anolyte composition.^19,40–42^

Average TN removal ranged from 95.1% ± 6.9% in MEC operation to 77% ± 13% in MFC mode (Figure 1B). TN removal from the anolyte is attributed to the transport of NH_4_^+^ across the CEM due to the electromigratory force created from the flow of electrons from the anode to the cathode. The importance of this electromigratory force for TN removal is highlighted in a study by Burns et al., where the same system was used to recover ammonia from orgN in real dairy manure and no TN removal from the anolyte was observed in conditions with no current flow between electrodes.^5^ Elevated TN removal in MEC operation can contribute to the increased electromigration force created by the additional applied potential difference between electrodes (*E*_appl._ = 0.8 V). TAN concentrations in the anolyte exhibit interesting trends in each operation mode. In the MEC mode, anolyte TAN concentration decreased in both replicate TS experiments, going from 100 to 95 mg N L^–1^ in MEC 1 and from 270 to 95 mg N L^–1^ in MEC 2 (Figure 1C). The elevated initial TAN concentration in MEC 2 is likely due to accumulation of TAN in the anolyte over the several maintenance cycles in between data collection for MEC 1 and MEC 2 (see Section 2.1.1), and is the reason for the large error measurement shown. Despite this accumulation of TAN, the BES was still able to reduce the TAN concentration in the anolyte to a relatively low (<100 mg L^–1^) concentration. Both the high initial TAN concentration and the effective reduction in TAN concentration over the course of MEC 2 are promising. Accumulation of TAN indicates continued microbial orgN mineralization to produce NH_4_^+^, which is the ultimate goal of the cultivated microbial community in the anode. Furthermore, efficient removal of TAN from the anode chamber eliminates problems associated with NH_4_^+^ toxicity to the microbes and incentivizes continuous TAN production. The high removal of TAN from the anode chamber in both MEC 1 and 2 indicates that the BES operated in MEC mode is capable of efficient TAN transport across the CEM to the cathode regardless of initial TAN concentration. The similar final TAN concentration in the anolyte in both MEC 1 and MEC 2 suggests that there is perhaps a lower boundary to which TAN transport can occur out of the anolyte.

In MFC mode, the trend for anolyte TAN concentration was reversed, exhibiting an increase in concentration from the initial to final measurements. For MFC 1, the anolyte TAN concentration increased from 90 mg to 165 mg N L^–1^ during the batch cycle, while in MFC 2, the anolyte TAN concentration increased from 100 mg to 135 mg N L^–1^. Despite this increase in anolyte TAN, a net reduction in anolyte TN was still observed in the BES (Figure 1). This is possible due to the nature of both the system and the nitrogen measurements. TN consists of orgN and TAN, as nitrate and nitrite are below the detection limit in our system. Initially, there is a high amount of orgN in the anolyte (as glutamic acid) and minimal TAN. Over the course of the experimental cycle, microbes are able to degrade orgN into NH_4_^+^, which increases TAN. This NH_4_^+^ can be transported across the CEM, resulting in a net decrease in TN in the anolyte over the course of the experimental cycle (as shown in Figure 1B). The NH_4_^+^ transport across the CEM is not 100% efficient, resulting in some of the NH_4_^+^ (which was previously orgN and not observed in the initial TAN measurement) remaining in the anolyte at the end of the experimental cycle. This is reflected by the increase in TAN concentrations in the anolyte from the initial to final measurements (Figure 1C). While this same process occurred in MEC operation mode, the decreasing TAN concentration indicates that the additional electromigratory force present due to the applied potential difference across the electrodes (0.8 V in MEC mode as opposed to no additional applied voltage in MFC mode) was able to enhance the NH_4_^+^ transport across the CEM.

The increasing trend in TAN concentration observed in MFC suggests that TAN transport is significantly reduced without the additional electromigratory force (as potential difference across electrodes) previously applied in MEC. This trend was also observed previously from the same system, where, when the reactor was fed with glycine in MFC operation, anolyte TAN trends fluctuated between cycles, sometimes exhibiting an increase from initial to final measurements and sometimes decreasing.^5,19^ The opposite trends observed in differing operation modes in this study can further be explained when accounting for the microbial community adaptation that occurred under different operation modes. In this study, the MEC operation mode was investigated first, over several experiments and maintenance cycles from late May to late October 2024 (∼150-day period). During this time, the microbial community in the anode chamber adapted to the externally applied potential difference and the corresponding increased electromigratory force, which served to draw additional TAN (as NH_4_^+^) out of the anode chamber across the CEM. This adaptation resulted in increased efficiency in N mineralization, as indicated by the combination of the superior TN removal and decreasing anolyte TAN concentration in the MEC operation mode. With the increased TAN removal in the MEC mode, the microbes could mineralize more TAN without reaching toxicity. After removing the externally applied potential difference, the BES (now in MFC operation mode) was run through several maintenance cycles over a period of 19 days before data for MFC 1 and 2 were collected continuously (e.g., back-to-back experiments, 96 h total). Although the COD and TN removal in the system was stabilized after 19 days, the microorganisms did not completely revert to pre-MEC operation N mineralization rates, as indicated by the excess TAN production in the anolyte, which accumulates due to the now decreased electromigratory force.

#### 3.1.2 Electrochemical Performance

Electrochemical performance metrics were recorded and calculated for each time series experiment, including the maximum current density (in A m^–2^ of cathode area), the Coulombic efficiency (CE) (eq 3), and the nitrogen loading ratio (*R*_N_) (eq 4). In MEC operation mode, average maximum current density was approximately 0.670 ± 0.015 A m^–2^, whereas in MFC operation mode, the average maximum current density was 1.157 ± 0.013 A m^–2^. This is in contrast with previously published studies, where MEC operation generally results in a higher current density due to the externally applied potential difference.^10,43^ CE is elevated in MFC operation at an average of 0.01177 ± 0.00084 compared to an average of 0.0069 ± 0.00031 in MEC operation (Figure 1D). Based on the calculation of CE (eq 3) and the reported differences in COD removal and maximum current density, it is apparent that the anodic microorganisms more effectively produce extracellular electrons during COD degradation to contribute to the current generation in the system in MFC operation mode. In MEC operation, the larger removal of COD from the anolyte increases the denominator of the CE fraction in eq 3; however, there is no corresponding increase in *Q* in the numerator in this operating condition. In fact, there is an observed decrease in maximum current density and *Q* in MEC operation, resulting in a lower overall CE. The opposite is true for experiments in the MFC operation condition. From these observations, it is apparent that microbes in the anode, while more effective at COD removal in MEC operation, are better at electron production from COD degradation and electron transfer to the electrode in MFC operation.

In both operation modes, the CE is quite low, particularly compared to that reported in other studies.^40,44,45^ The high COD removal coupled with a very low *Q* point to potential inefficiencies in current generation and transfer within the BES. These inefficiencies could arise as a result of any number of physical and operational parameters, such as inappropriate discharge to other equipment (“short-circuiting”) or charge loss and potential drop through the measurement instrument. It is also important to note that a large portion of the COD present in the synthetic manure substrate is from the orgN compound, glutamic acid, which is roughly 2.5× more concentrated in the anolyte than the carbon-based organic compound, acetate. While there is ample published work indicating the effectiveness of exoelectrogens using acetate as an electron donor, there is limited work indicating how, if at all, these or other organisms can generate the free electrons contributing to *Q* (and therefore CE) measurements using orgN.^40,45^ Furthermore, many of the known pathways for glutamic acid degradation are fermentative, which would not produce extracellular electrons to contribute to current generation.^46,47^ As such, further investigation into the electrogenic metabolism using an orgN substrate is warranted.

In MEC operation mode, the nitrogen loading ratio (*R*_N_), a measure of how efficiently current production is used to drive N transport out of the cathode (eq 4), averaged 7.92 ± 0.80 mol N mol^–1^ electrons. In MFC operation mode, *R*_N_ decreased, with an average of 4.04 ± 0.84 mol N mol^–1^ electrons (Figure 1E). This finding is consistent with the previously reported differences in TN removal and anolyte TAN trends in the different operating conditions. Like the interplay between changes in COD removal and *Q* in the different operation conditions, TN removal and *Q* combine in the *R*_N_ fraction in eq 4. In MEC operation, the decreased *Q* in the denominator combined with the greatly increased TN removal in the numerator results in an overall larger *R*_N_, whereas in MFC operation, the increased *Q* and decreased TN removal result in an overall smaller *R*_N_. This finding may indicate that orgN removal, which is assumed to occur as part of the calculated TN removal since microbes have to first mineralize orgN to NH_4_^+^ for it to be transported out of the anode and measured in the TN removal, does not contribute strongly to current production and total change transferred in the system. This assertion is also supported by the aforementioned low CE despite high COD removal, considering that a large portion of the COD in the system was the orgN compound.

The calculated *R*_N_ in both operation conditions is fairly high, and much higher than previous measurements with this system operating as an MFC treating both synthetic and real dairy manures.^5,19^ This is likely due to a combination of the simpler synthetic substrate and the significant adaptation time permitted for the system, both before and during this study.^5,19^ Previously, this system was operated for ammonia recovery from synthetic manure containing glycine as an orgN source.^19^ Despite the simplicity of the glycine (compared to the glutamic acid used as an orgN source in this study), the system had not been operating to mineralize orgN for very long, and thus the study yielded lower *R*_N_ values, between less than 0.5 and just over 2 mol N mol^–1^ electrons.^19^ When the same system was used to recover ammonia from real dairy manure substrates, the *R*_N_ values varied between just over 0.5 and 1.1 mol N mol^–1^ electrons.^5^ The relatively low values here are attributed to the complexity of the dairy manure substrates. In this study, the combined effects of prolonged system operation over several years (allowing the microbial community and system to adapt to become very efficient at both orgN mineralization and NH_4_^+^ transfer to the catholyte) and a simpler substrate (compared to the dairy manures used previously) likely led to the record-high *R*_N_ values. Furthermore, the composition of the catholyte, which contained concentrated Na^+^, could have influenced NH_4_^+^ transport through Donnan dialysis (DD). DD is a membrane process that allows for the exchange of ions of like-charges opposite the concentration gradient.^48^ In this case, Na^+^ ions in the catholyte can exchange with NH_4_^+^ ions in the anolyte to enhance transport of TAN out of the anolyte, thus leading to an elevated R_N_ by increasing the numerator in eq 4. In a series of measurements from the same system prior to this experimental work, the catholyte Na^+^ concentration was observed to decrease from 251 to 216 mg L^–1^, and the anolyte Na^+^ concentration was seen to increase from 1509 to 1636 mg L^–1^, indicating minor DD effects. The enhancement effects of DD on TAN recovery from manure have been studied in detail elsewhere.^49^

### 3.2 Time Series Analysis

As previously described in Section 2.2.1, two replicate time series experiments were run under each operation condition. Roughly 7 samples of anolyte solution were taken over the course of each TS, and each sample was analyzed for COD, TN, TAN, and acetate concentrations, as well as used in Raman analysis.

#### 3.2.1 COD, Acetate, TN, and TAN Concentrations

For all TS under both operation conditions, the COD and TN concentrations in the anolyte closely follow the recorded current density (Figure 2). In all TS experiments, COD and acetate concentrations drop to nearly zero within the first 24 h of the cycle and remain at or near zero for the remainder of the experiment. The recorded current density follows a similar trend, increasing initially with the fresh substrate (indicating increased microbial degradation activity while food is available) and slowly decreasing over the first 24 h of operation time as substrate is consumed.

**Figure 2:**
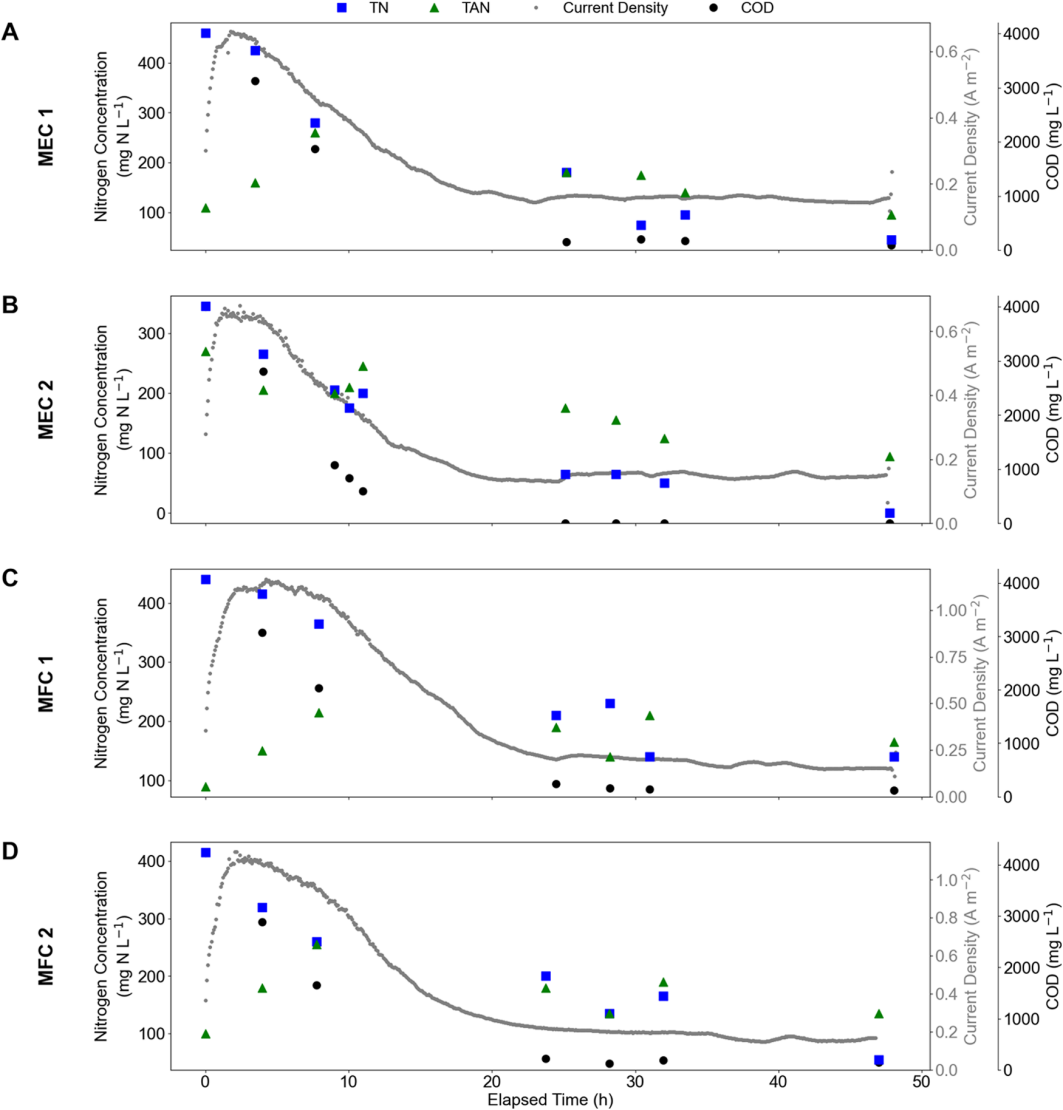
COD and N concentrations and current density in time series experiments MEC 1 (A), MEC 2 (B), MFC 1 (C), and MFC 2 (D). COD concentrations are reported in the secondary right-hand *y*-axis, and total and total ammoniacal nitrogen (TN and TAN, respectively) concentrations are reported in the left-hand *y*-axis. In all panels, the gray points in the background show the recorded current density on the primary right-hand *y*-axis at 5 min intervals over the duration of the TS experiments.

TN also exhibits a decreasing trend over the duration of each TS experiment, although the slope of the decrease is more gradual, with degradation continuing well into the second day of cycle operation, particularly in the MFC operation (Figure 2). The quick decrease in COD concentration in all TS is indicative of the relative simplicity of the substrate for the microbes to degrade. The decreasing overall COD concentration, in conjunction with the swift decrease in acetate concentration, shows that both the organic carbon and nitrogen (glutamic acid) sources are easy for the microbes to degrade. The slight decrease in TN concentration is indicative of the interplay between orgN degradation and actual removal from the anolyte, which must happen as NH_4_^+^ transport across the CEM. The TS data suggest that the microbes effectively degrade orgN, resulting in the rapid decrease in COD, but the transport of mineralized orgN (as NH_4_^+^) across the CEM limits observed TN removal. This finding suggests that TAN transport across the CEM is the rate-limiting step as opposed to orgN mineralization, which is the opposite of findings previously published when the substrate is real dairy manure.^5^ This difference is expected, as with real dairy manure, the orgN substrate is much more complex and requires more time, energy, and metabolic resources for microbes to degrade. This has important implications for future work regarding BES as TAN recovery technologies through orgN mineralization, as practitioners will need to consider whether transport or mineralization will be rate-limiting to the process, depending on the complexity of the manure substrate.

Total ammoniacal nitrogen (TAN) concentrations in the anolyte in most TS exhibit a characteristic increasing-then-decreasing trend (Figure 2). The initial increase in TAN concentration, in combination with the decrease in COD in each experiment, supports the presence of orgN mineralization reactions. As microbes degrade the orgN, the COD concentration decreases and TAN increases as NH_4_^+^ is produced. In the early stages of each experiment, orgN mineralization rates exceed the rate of TAN transport out of the anode chamber and hence the increase in TAN concentrations. Between 10 and 24 h of operation, the orgN mineralization rate slows, likely due to the decreasing concentration of orgN available for microbes to degrade as the experiment continues. At this point, TAN transport out of the anode chamber starts to exceed TAN production, resulting in an observed decrease in the TAN concentration in the anolyte solution. In MFC 1 and MFC 2, the TAN concentration in the anolyte does not achieve a level below that of the initial concentration, indicating that there was not enough time or electromotive force to complete the removal of produced TAN from the anolyte (see Section 3.1.1). However, it is important to note that the TN concentration steadily decreases throughout the duration of all of the TS experiments, supporting BES in both MEC and MFC operation modes as successful N removal and recovery mechanisms.

#### 3.2.2 Raman OrgN Analysis

Samples from each TS experiment were analyzed by using Raman spectroscopy to characterize the orgN content in the BES throughout the duration of each cycle. Results from preliminary Raman analyses of pure, concentrated, and mixed composition orgN samples revealed three distinct Raman bands to track (Figure 3). In Figure 3, “Origin” indicates the spectra obtained from Raman analysis of a solution of pure, concentrated (7.7473 g L^–1^) glutamic acid, “Pristine” indicates the spectra obtained from Raman analysis of a sample of the synthetic manure solution (glutamic acid, acetate, and other trace elements, see Section 2.1.2) before mixing with the retained anode chamber solution (see Section 2.1.1 for a description of the solution change procedure), and “Initial Anolyte” indicates the spectra obtained from Raman analysis of a sample of the initial anolyte solution after mixing. The band intensities at 915, 1397, and 2931 cm^–1^ emerged as dominant in each spectrum, and as such, were chosen as bands characteristic of glutamic acid to track as a proxy for glutamic acid concentration (Figure 3).

**Figure 3:**
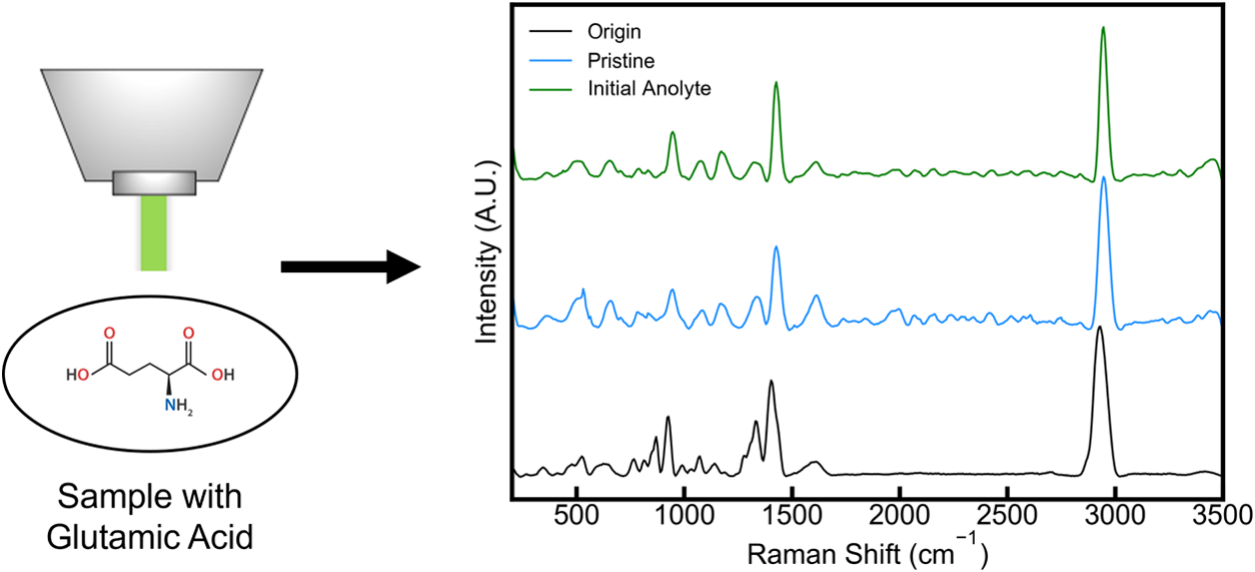
Raman spectra from three samples containing glutamic acid with characteristic bands for glutamic acid (915, 1397, and 2931 cm^–1^) indicated by dashed lines. “Origin” denotes a sample of pure glutamic acid in DI water, “Pristine” denotes a sample of synthetic manure anolyte (containing glutamic acid, acetate, and other trace compounds, see Section 2.1.2) before mixing with anolyte previously treated by the reactor (see Section 2.1.1), and “Initial” denotes a sample of synthetic manure anolyte after it has completely mixed with remaining reactor anolyte for 10 min at the start of an experimental cycle (see Section 2.1.1).

The band intensity in each sample was plotted against the elapsed time within the experiment and compared to the current density, as shown in Figure 4. For all TS in both operation conditions, the intensity of the 2931 and 1397 cm^–1^ bands appears to most closely follow decreases in current density and COD and TN concentrations. This observation, in conjunction with the spectra presented in Figure 3, supports the conclusion that the 2931 and 1397 cm^–1^ bands are sufficient markers for glutamic acid concentration. Significant further method development is required to determine which bonds within the glutamic acid molecule each of these bands indicates, as well as to refine the measurement to be more consistent and to determine a correlation that will allow for the calculation of exact concentrations of glutamic acid.

**Figure 4:**
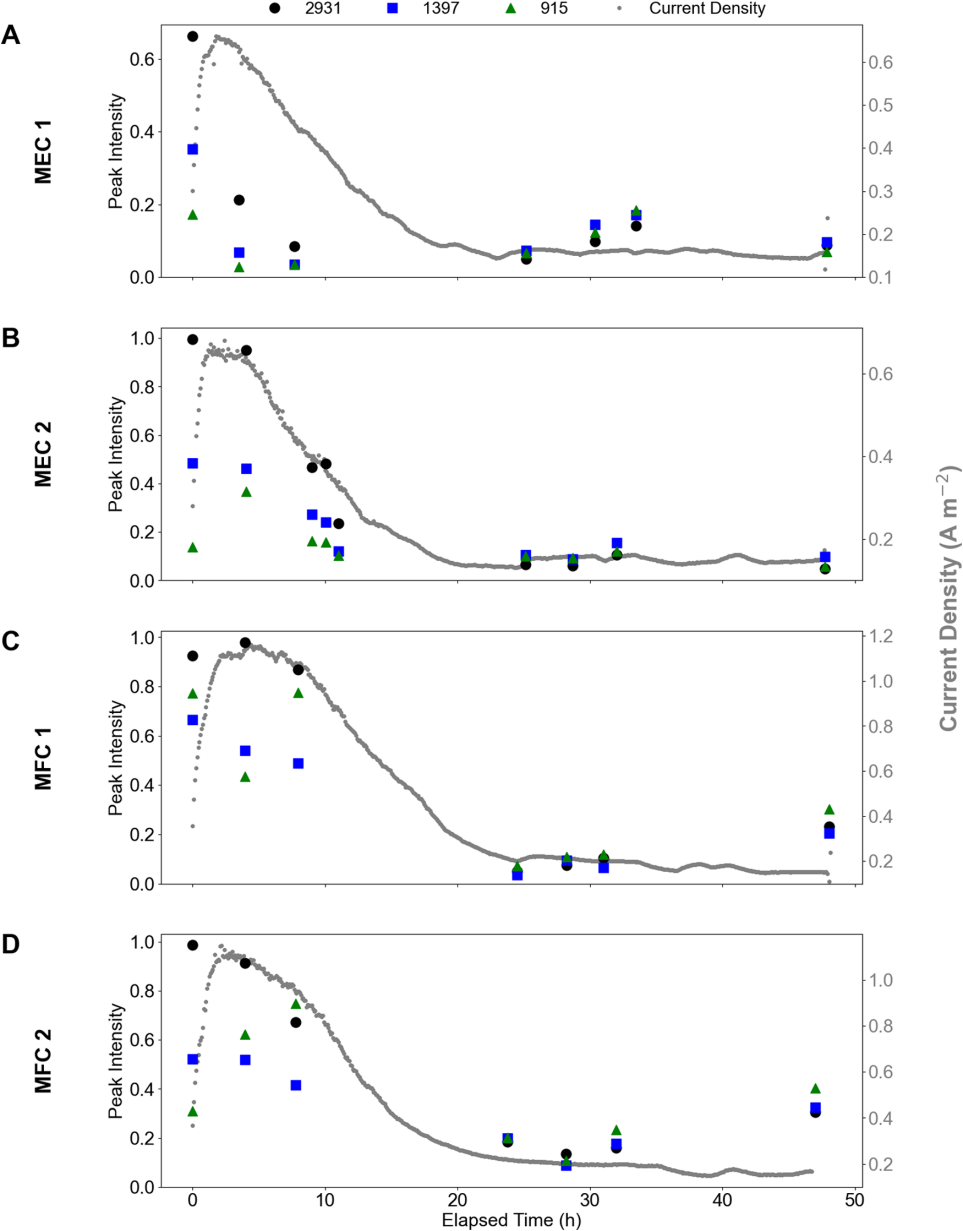
Raman band intensity and current density over the course of each time series experiment: MEC 1 (A), MEC 2 (B), MFC 1 (C), and MFC 2 (D). In all panels, the gray data points in the background show the recorded current density at 5 min intervals over the duration of the TS experiments. Current density is plotted on the secondary *y*-axis.

The 915 cm^–1^ band does not agree with either of the other Raman bands selected or with the COD, TN, or current density data collected. The 915 cm^–1^ band does, however, exhibit some similarities to the trends observed with the TAN concentration in the TS samples. While measurement of NH_3_ and NH_4_^+^ in aqueous solutions with Raman is challenging, it has been demonstrated with some success elsewhere, and other studies have shown bands at 934 and 967 cm^–1^ when analyzing gaseous NH_3_ concentrations.^50,51^ We speculate that the bond indicated by the 915 cm^–1^ band could be associated with NH_3_, NH_4_^+^, a chemical precursor to NH_4_^+^ in the mineralization of glutamic acid, or another product from glutamic acid degradation. Depending on the specific pathway, chemical precursors could be compounds like methylaspartate, mesaconate, and citramalate (characterizing the β-methylaspartate fermentation pathway for anaerobic degradation of glutamate) or ketoglutarate, hydroxyglutamate, and crotonyl-CoA (characterizing the hydroxyglutarate fermentation pathways).^46,52^ Other glutamate degradation pathways may produce oxaloacetate, fumarate, and succinyl-CoA.^47^ Final products from glutamate degradation that may respond to Raman probing could be butyrate, acetate, and propionate, although the low recorded acetate concentrations in this work suggest that any acetate produced in glutamate degradation is quickly scavenged by acetoclastic exoelectrogens in the anode chamber (see Section 3.3).^53^ As such, further investigation of the exact bond allocation of each of the selected bands is necessary in future method development in this application of Raman analysis.

Another interesting observation is the increase in intensities of all bands in the second half of the TS experiments in the MFC operation conditions (Figure 4C,D). This increase is perplexing, especially considering there is no observed corresponding increase in TN concentration. There is, however, a slight increase in COD concentration observed toward the end of each experiment in MFCs 1 and 2. The exact reason behind the increased intensities and COD concentrations requires further investigation; however, one possibility is that some attached growth from the anode brush was resuspended through agitation of the reactor during sampling. This increase is reminiscent of increasing orgN concentrations reported previously in a study by Burns and Qin^19^ during ultralow concentrations of orgN, where premature cell death and lysis were inferred to occur due to a lack of easily degradable orgN concentration for microbes. Further study of the microbial community and metabolisms within the reactor would reveal more information and conclusions regarding this observation.

### 3.3 Degradation Kinetics

Data from time series experiments was used to evaluate degradation kinetics for COD, TN, acetate, and orgN (by proxy of Raman band intensities). Unmodified and modified (natural log of concentration, inverse of concentration) plots of concentration or band intensity versus time were created for each constituent (Section 2.2.4). Linear regression was performed on each set of data (unmodified and any modified data sets), and an *R* ^2^-value was calculated. A minimum *R* ^2^ of 0.85 was used as the basis for assuming acceptable linearity, with a few exceptions as discussed in the following sections. In the case that no data sets (modified or unmodified) for a single constituent yielded acceptable linearity, the constituent’s kinetics were deemed undeterminable within the scope of the current study.

Linear regression on unmodified plots of COD vs time for all TS experiments yielded moderate R-squared values ranging from 0.6 to 0.8, requiring further analysis to determine the order of reaction (Figures S3, S14, S25, and S36). When evaluated logarithmically (i.e., natural log of COD concentration versus time), COD degradation indicated first-order reaction kinetics (Figure 5A). MFC 2 showed the weakest correlation with an *R* ^2^-value of 0.85, whereas MFC 1 demonstrated the best fit with an *R* ^2^ of 0.98. The lower *R* ^2^-value for MFC 2 could be explained by an unusual increase in COD concentration toward the end of the TS experiment, creating an outlier on the plot. MEC 1 and MEC 2 exhibited strong correlations, with *R* ^2^-values of 0.92 and 0.95, respectively. Based on these analyses, COD degradation in both fuel cell and electrolysis cell operation is deemed first order, with rate constants ranging from 0.087 h^–1^ in MEC 1 to 0.174 h^–1^ in MEC and 0.083 h^–1^ in MFC 2 to 0.098 h^–1^ in MFC 1 (Table S1). TS in the MFC condition exhibited the most varied linearity (indicated in a larger range of *R* ^2^-values), indicating that kinetics for COD degradation in MFC operation are less stable than in MEC operation.

**Figure 5:**
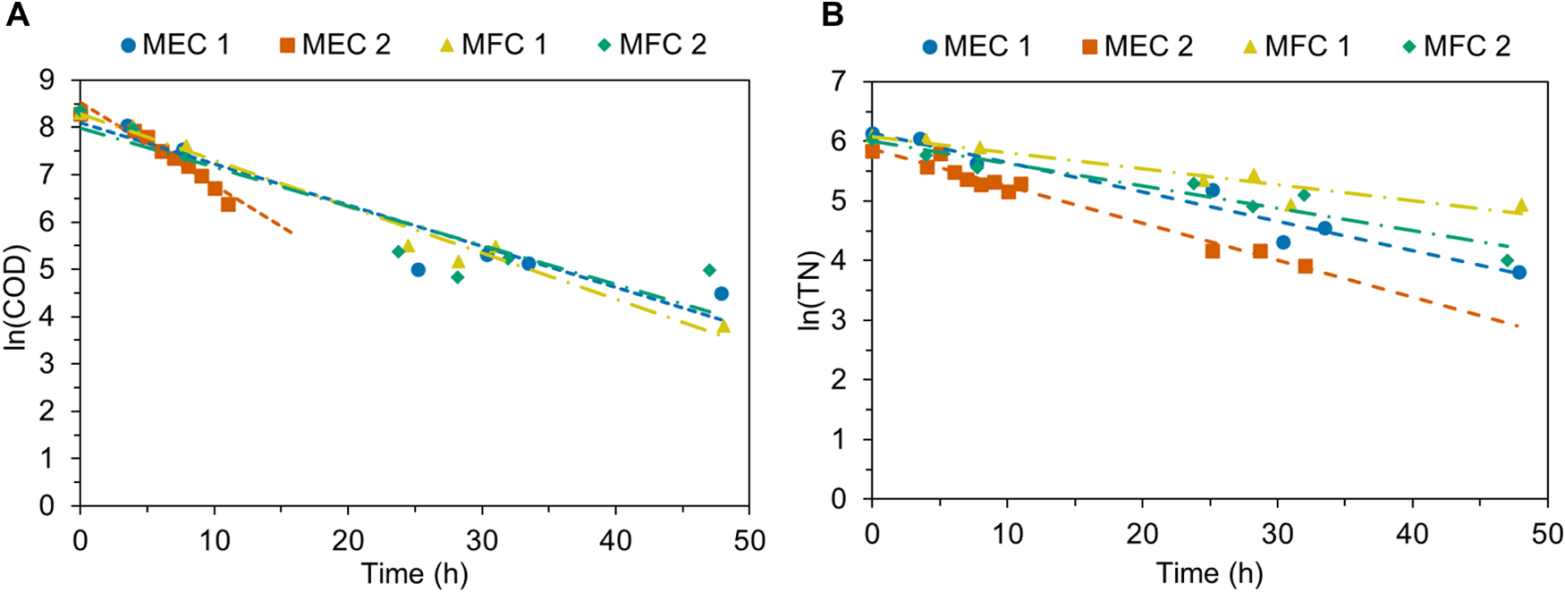
First-order kinetics for COD (A) and TN (B) degradation in the BES. Linearization of experimental data was accomplished through logarithmic manipulation of the concentrations for both constituents. Trendline fits can be found in Tables S1 and S2. Experimental data are indicated as markers, and fitted trendlines are shown as dashed lines, with MEC operation being steady dashes and MFC operation being dot-dashes. Note that MEC 2 exhibited complete degradation of COD after 11 h and complete degradation of TN after 30 h, and zero values are not shown.

Kinetic analysis of TN degradation throughout each TS experiment yielded interesting results. For all experiments, unmodified plots of TN concentration versus time yielded seemingly acceptable linearity, with *R* ^2^-values ranging from 0.87 to 0.92 (Figures S4, S15, S26, and S37). However, visual inspection of unmodified data shows significant variation from linear trends, and more closely resembles a gradual exponential decay (Figures 2, S4, S15, S26, and S37). This is confirmed in plotting the natural logarithm of TN concentration versus time for each TS experiment, which reveals superior linearity with *R* ^2^-values increasing for each TS experiment from those obtained in the regression on unmodified data (Figure 5B). MEC operations yielded *R* ^2^-values of 0.96 and 0.98 for MEC 1 and MEC 2, respectively, and MFC operation resulted in *R* ^2^-values of 0.92 for both MFC 1 and 2 (Table S2). From these analyses, we conclude that TN degradation likely follows a first-order degradation reaction in the BES. Reaction rate coefficients for TN degradation were slightly more varied between operation conditions than those for COD degradation, ranging from 0.049 h^–1^ in MEC 1 and 0.062 h^–1^ in MEC 2 to 0.028 h^–1^ in MFC 1 and 0.037 h^–1^ in MFC 2 (Table S2). This variation is explained by the impact of the externally applied potential in MEC operation; the *E*_appl_ of 0.8 V between electrodes in MEC operation resulted in increased TN removal, therefore incentivizing increased kinetics of TN degradation. Previous studies concerning nitrogen removal from wastewater with MECs equipped with cation exchange membranes concluded that catholyte NH_4_^+^-N increased remarkably when current generation increased.^54^

Acetate reaction order kinetics remained undetermined due to a lack of sufficient data points throughout the TS experiments, considering that acetate concentration drops to zero within the first 10 h of experimental operation (Figures S5, S16, S27, and S38). From this, we conclude that in both MEC and MFC operation modes, acetate is degraded efficiently and first as the easiest substrate available for the microbes in the anode chamber. Furthermore, any additional acetate created in the degradation of the glutamic acid is immediately scavenged by other microbes in the anode compartment. Plotting logarithmic and inverse acetate concentration versus time was also inconclusive, with *R* ^2^-values for logarithmically transformed data ranging from 0.22 to 0.84 (Figures S6, S17, S28, and S39) and those for inverse of acetate concentration ranging from 0.19 to 0.89 (Figures S7, S18, S29, and S40). Notably, in MEC 2, increased sampling resolution was used to try to elucidate the acetate degradation kinetics within the first 10 h of experimental operation before concentrations dropped to zero. While both the logarithmically transformed and the inversely transformed data for acetate concentration versus time in MEC 2 have relatively high *R* ^2^-values (0.82 and 0.89, respectively), visual inspection of these plots clearly indicates nonlinear relationships (Figures S17 and S18). This finding, much like the discrepancies between acceptable *R* ^2^-values and visual inspection in the TN kinetic analysis, indicates some of the limitations of this analysis and further highlights the need for a more precise approach. Interestingly, while quantifying acetate concentration in all TS experiments via high-performance liquid chromatography, we noticed an unidentified peak which coelutes with acetate as a leading peak, increasing in intensity as acetate peak intensity decreases before dropping to zero at the same time acetate concentrations decrease to zero (Figure S2). This peak, while unidentified in this study, could be a product of glutamic acid degradation and is worthy of further investigation and attempts at identification, such as through liquid chromatography mass spectrometry.

Finally, orgN degradation kinetics was also deemed inconclusive within the analysis of this study. *R* ^2^-values for unmodified band intensity plotted versus time in MEC 1 were 0.36 and 0.061 for bands 2391 cm^–1^ and 1397 cm^–1^, respectively (Figures S8 and S11). Both logarithmic and inverse analyses further resulted in inconclusive kinetics, having *R* ^2^-values ranging from 0.21 to 0.35 for the 2931 cm^–1^ band (Figures S9 and S10) and 0.0005 to 0.066 for the 1397 cm^–1^ band (Figures S12 and S13). MEC 2 showed similar trends with *R* ^2^-values for unmodified band intensity versus time equal to 0.69 for the band 2931 cm^–1^ and 0.59 for the band 1397 cm^–1^ (Figures S19 and S22). Logarithmic and inverse analyses for the 1397 cm^–1^ band showed little improvement, with *R* ^2^-values ranging from 0.65 to 0.75 (Figures S23 and S24). However, both logarithmic and inverse analyses of the 2931 cm^–1^ band versus time for MEC 2 indicated acceptable linearity, with *R* ^2^-values of 0.90 and 0.91, respectively (Figures S20 and S21). Since this result was unable to be replicated in previous and subsequent experiments (data not shown) and given that visual inspection of the plots reveals significant variation from linear trends, we conclude that this linearity is coincidental and not scientifically indicative of singularly ordered (first or second) reaction kinetics for orgN degradation in the system. Unmodified and modified band intensity tracking for the TS samples collected in the MFC 1 and MFC 2 were also erratic, with *R* ^2^-values for the 2931 cm^–1^ band ranging from 0.21 to 0.71 (across unmodified, logarithmic, and inverse linearization) and between 0.15 and 0.64 for the 1397 cm^–1^ band (across unmodified, logarithmic, and inverse linearization) (Figures S30–S35 and S41–S46). Based on these analyses, we conclude that the kinetics of orgN degradation in BES as measured using Raman spectroscopy are zero-, first-, and second order. Inconclusive kinetic ordering could be explained by the lack of refinement in this application of Raman technology, or could indicate that orgN degradation cannot be singularly ordered within these systems. Given the propensity of microbial systems to follow Monod-type degradation kinetics, further investigation should consider this model applied to orgN degradation in BES, as well as pursue explicit quantification of orgN concentrations.^27,55^ Despite the undetermined kinetics, Raman analyses proved to be useful for showing generalized orgN content trends (Figure 4) and show promise as an orgN detection and quantification method with proper process development and refinement.

## 4 Conclusions

In this study, we investigated degradation kinetics for organics and N in configurations of BES for the recovery of NH_3_ from synthetic dairy manure. Glutamic acid, an amino acid found in high concentrations in dairy manure, was used as a synthetic organic nitrogen source, and the BES was operated in both electrolysis cell and fuel cell modes. Time series (TS) experiments were conducted with samples collected throughout the experiments for measurement of COD, TN, TAN, and acetate concentrations. Samples were also analyzed with Raman spectroscopy, with the intensities of the 2931 and 1397 cm^–1^ bands used as proxies for orgN content. MEC operation was found to enhance N removal from the anolyte, with an average of 95.1% ± 6.9% removal of TN and a net decrease in anolyte TAN concentrations in both TS experiments. Enhanced N removal in MEC mode is further supported by the superior reaction kinetics calculated for TN removal, which followed a first-order reaction (*R* ^2^ ≥ 0.96 for both TS experiments) with rate coefficients ranging from 0.05 to 0.06 h^–1^. Microbes were found to be more efficient at generating current in MFC operation, as indicated by the elevated Coulombic efficiency (CE) of 0.01177 ± 0.00084 and maximum current density of 1.157 ± 0.013 A m^–2^ recorded in this operation mode. Raman spectroscopy was proven to be an effective method for identifying glutamic acid in samples of mixed composition. The Raman analysis of samples from TS experiments indicated that the kinetics of glutamic acid degradation within the BES are complex and likely multiordered, as the data did not yield acceptable linearity for any iteration of unmodified or modified analysis.

The insights generated by this study are useful in guiding future research on BES as manure treatment and NH_3_ recovery systems. Further investigation and development of Raman spectroscopy as a method for detecting and quantifying orgN concentrations in complex samples could help to elucidate the orgN degradation kinetics within these systems. Furthermore, using Raman for such analyses will allow for simple, nondestructive investigation of sample composition, allowing for a streamlined approach to manure nutrient identification and quantification. Based on the aggregate and TS-specific data presented in this study, we can recommend utilizing MEC operation in systems targeting the removal and recovery of N from livestock manures. However, it is important for future research to consider the impacts of prolonged applied potential at the industrial level on aspects such as fuel consumption, energy costs, and greenhouse gas emissions. Results from this study indicate that intermittent MEC operation may be capable of accomplishing the same elevated N mineralization rates observed in continuous MEC operation, saving energy consumption. Overall, BES promises to contribute a sustainable solution focused on the circularity of nutrients to the problems created by increasing dairy herd size and manure production.

## Supplementary Material



## Notes

Trendline fits for COD and TN kinetic data as shown in Figure 5 in the text (Tables S1 and S2), COD removals for the 4 TS experiments (Figure S1), acetate and unidentified peak height over the duration of selected TS experiments (Figure S2), and linearizations and regressions for COD, TN, and Raman band intensities (as proxy for orgN) for each of the TS experiments (Figures S3–S46) (PDF)
