# Supplementary material for: Degradation Kinetics for Organic Nitrogen in Bioelectrochemical Systems toward Ammonia Recovery
Source: ACS ES T Eng. 2026 Mar 31;6(4):1369–81. doi: 10.1021/acsestengg.6c00128 (PMC13077639; doi:10.1021/acsestengg.6c00128)
Supplement: Supplementary file 1 [file ee6c00128_si_001.pdf]

Supporting Information for

DEGRADATION KINETICS FOR ORGANIC NITROGEN IN BIOELECTROCHEMICAL SYSTEMS TOWARDS AMMONIA RECOVERY

McKenzie Burns, Ziyan Wu, Tia Mirsha, Andrew Beaudet, Katie Mangus, and Mohan Qin*

Department of Civil and Environmental Engineering, University of Wisconsin–Madison, Madison, Wisconsin 53706, USA

*Corresponding author. E-mail: mohan.qin@wisc.edu

Table S1. Trendline fits for COD kinetic data shown in Fig. 5A.

Operation Mode	Trendline Equation	R^2-value
MEC 1	y = -0.0872x + 8.11	0.92
MEC 2	y = -0.1740x + 8.52	0.949
MFC 1	y = -0.0977x + 8.28	0.976
MFC 2	y = -0.0827x + 7.99	0.85

Table S2. Trendline fits for TN kinetic data shown in Fig. 5B.

Operation Mode	Trendline Equation	R^2-value
MEC 1	y = -0.0492x + 6.14	0.955
MEC 2	y = -0.0621x + 5.87	0.975
MFC 1	y = -0.0277x + 6.30	0.917
MFC 2	y = -0.0373x + 6.00	0.92

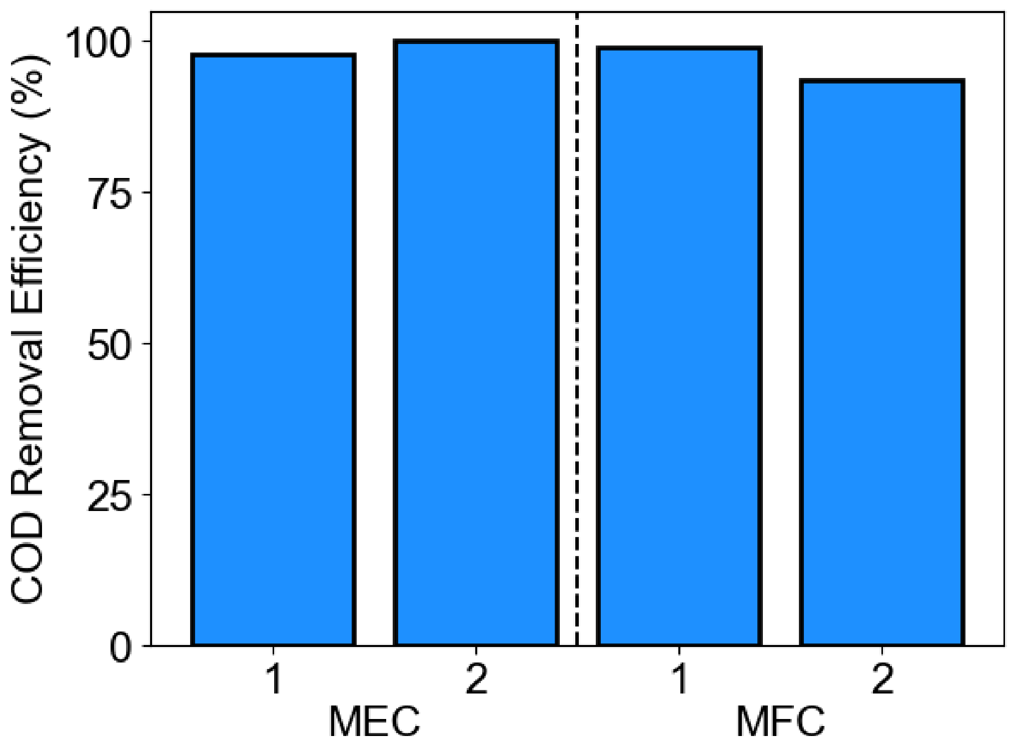

Figure S1. COD removal in TS experiments

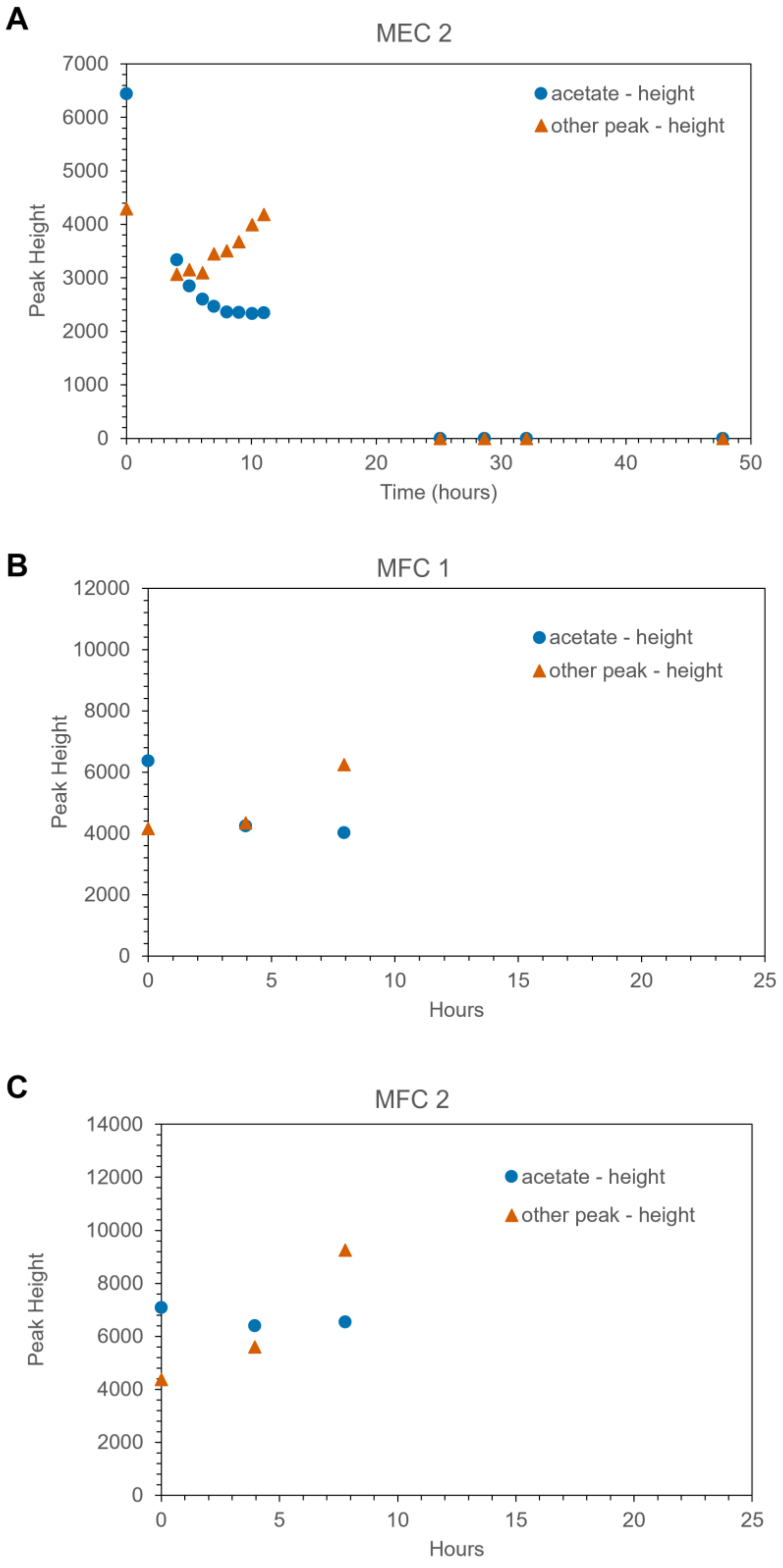

Figure S2. Acetate and unknown peak height over the duration of TS experiments.

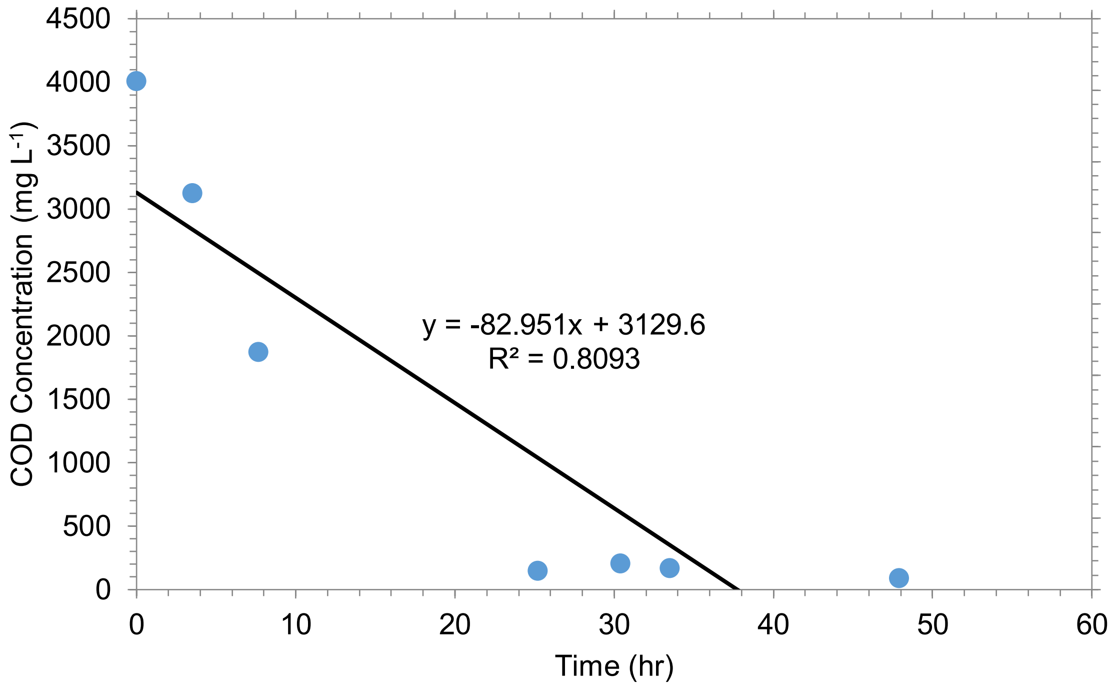

Figure S3. COD concentration vs time and linear regression for TS C.

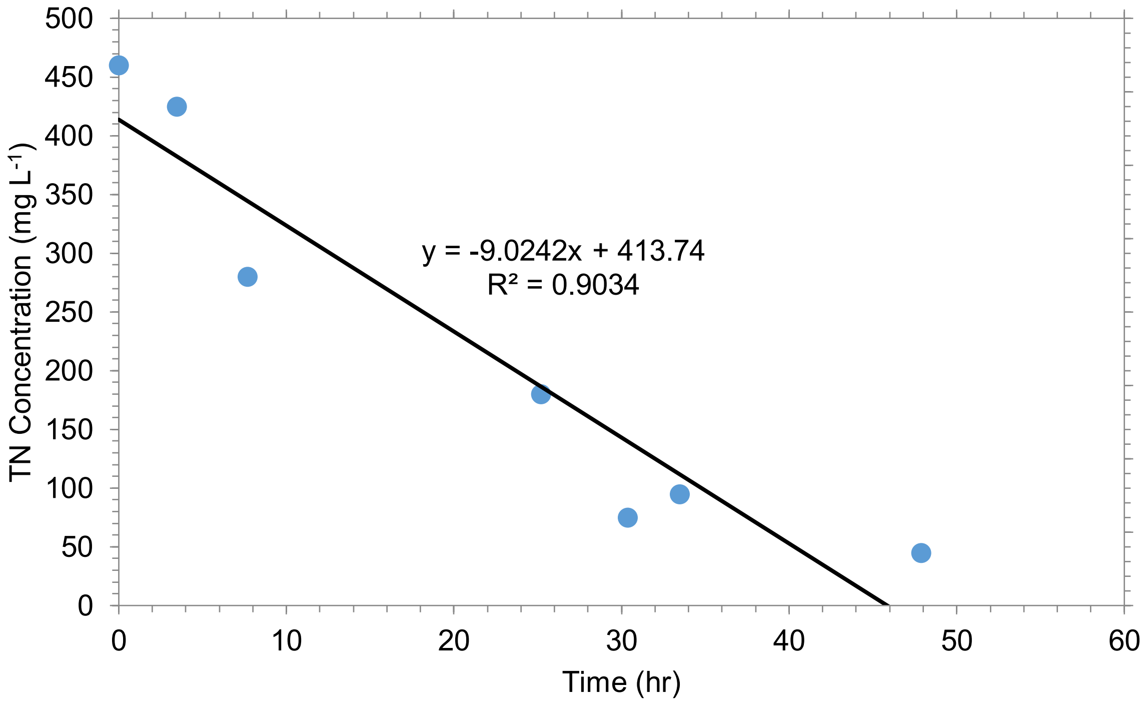

Figure S4. TN concentration vs time and linear regression for TS C.

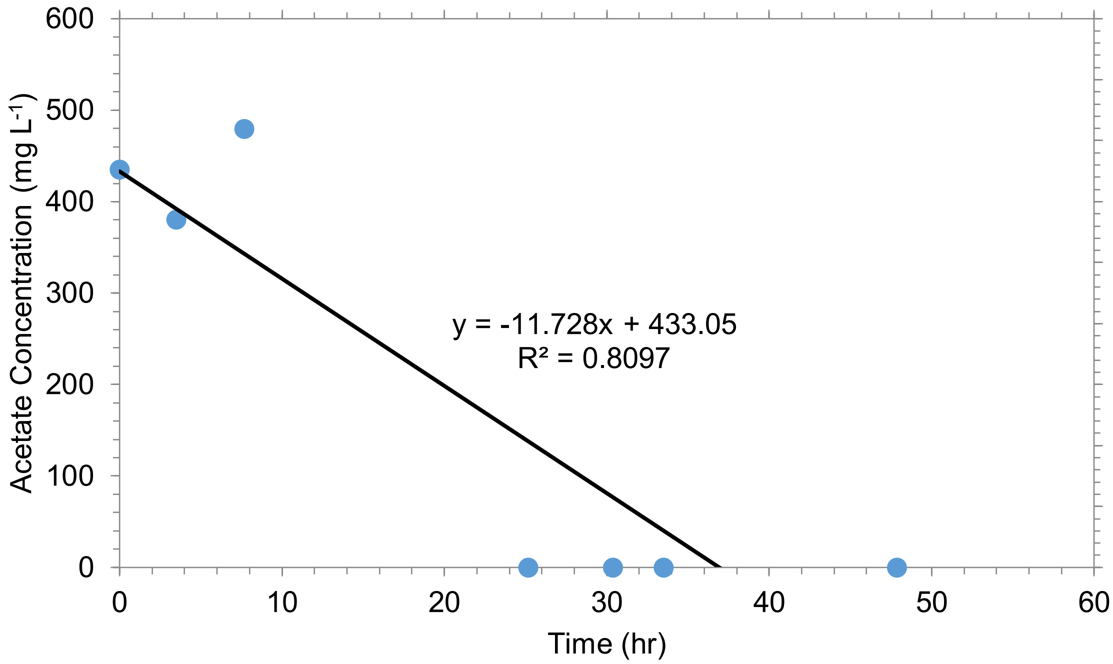

Figure S5. Acetate concentration versus time and linear regression for TS C.

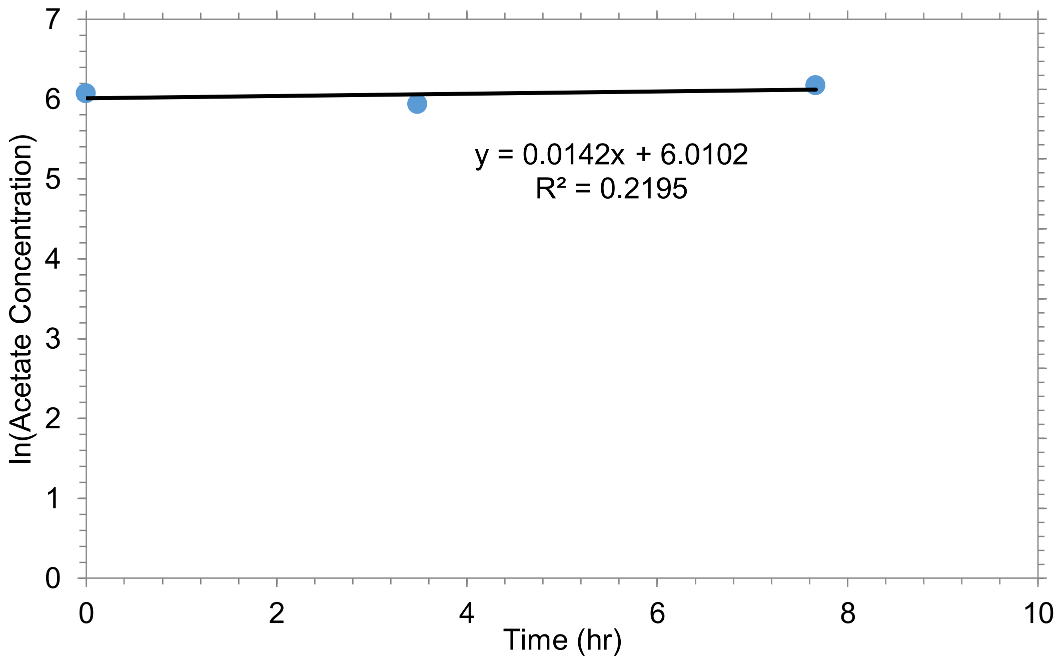

Figure S6. Natural logarithm of acetate concentration versus time and linear regression for TS C.

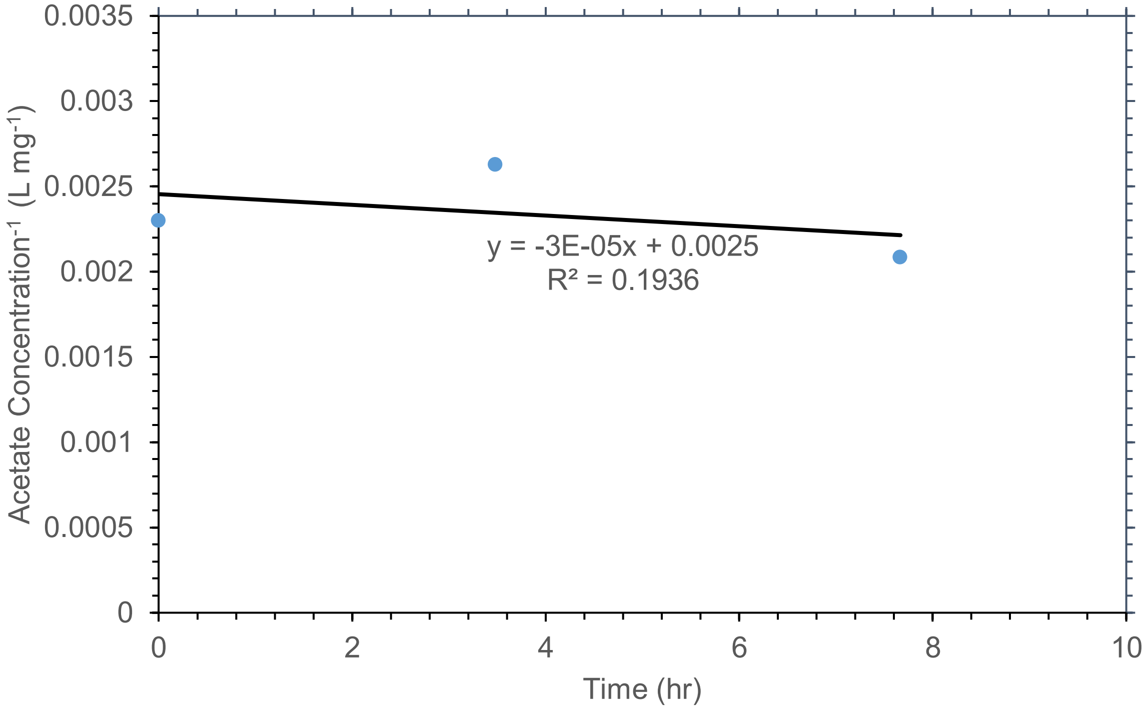

Figure S7. Inverse of the acetate concentration and linear regression for TS C.

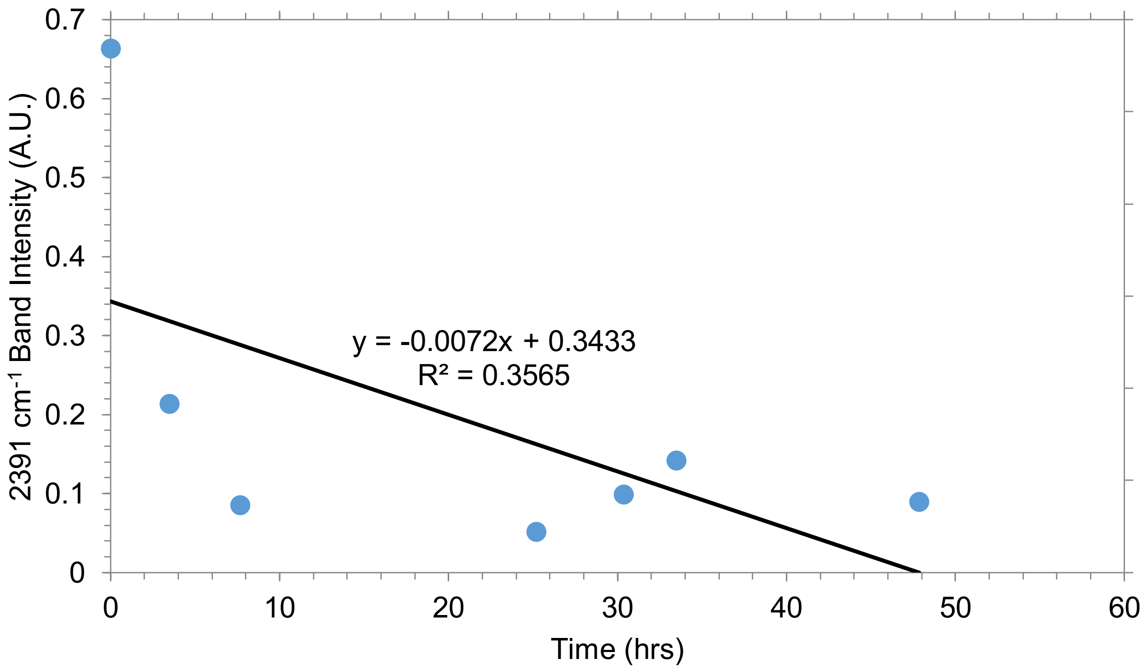

Figure S8. 2931 cm^{-1} band intensity versus time and linear regression for TS C.

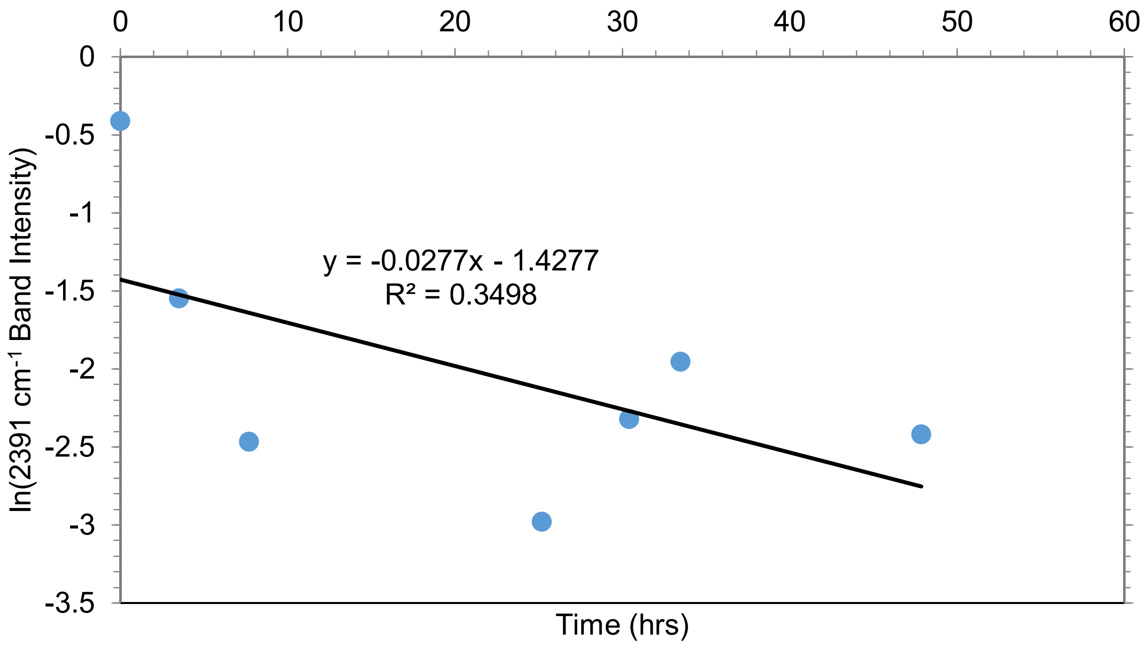

Figure S9. Natural logarithm of 2931 cm^{-1} band intensity versus time and linear regression for TS C.

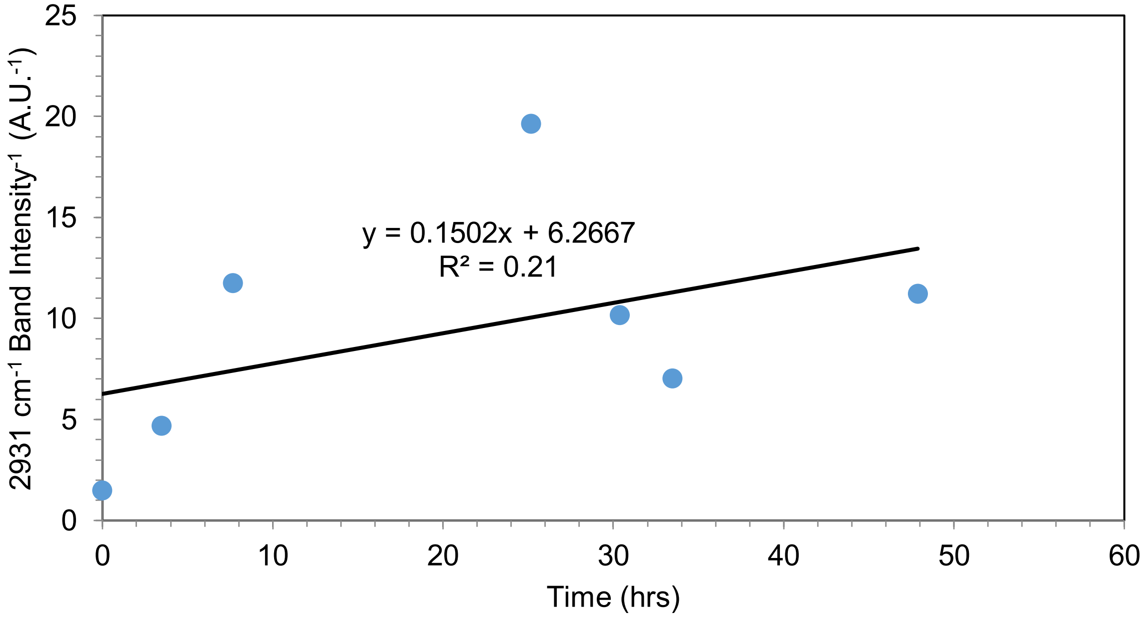

Figure S10. Inverse of 2931 cm^{-1} band intensity versus time and linear regression for TS C.

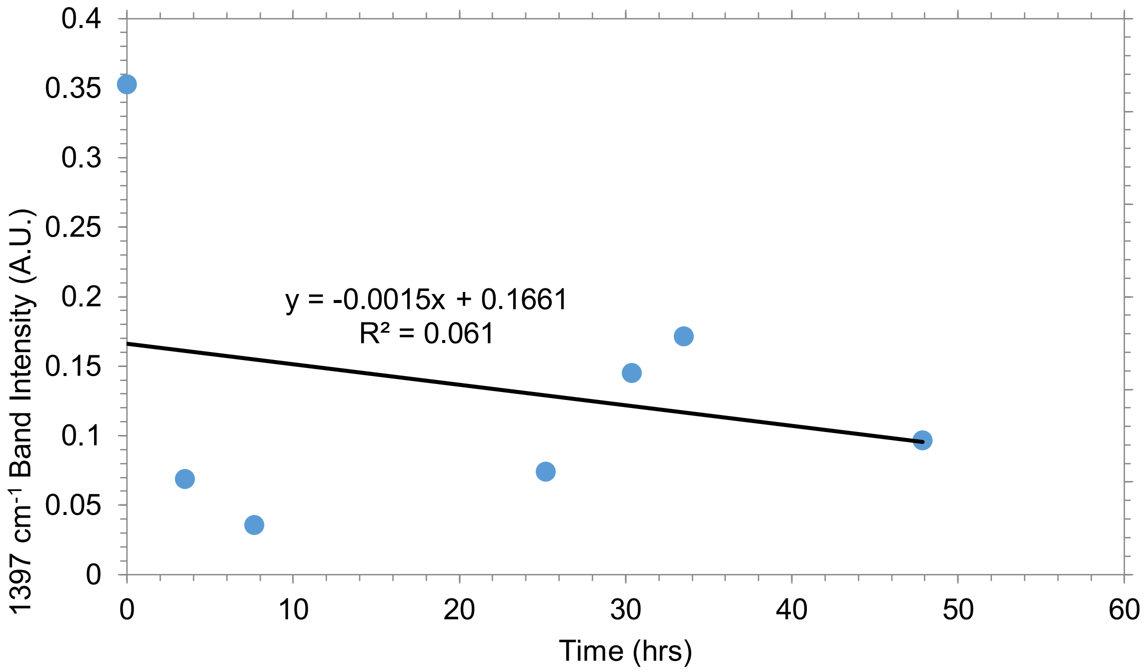

Figure S11. 1397 cm^{-1} band intensity versus time and linear regression for TS C.

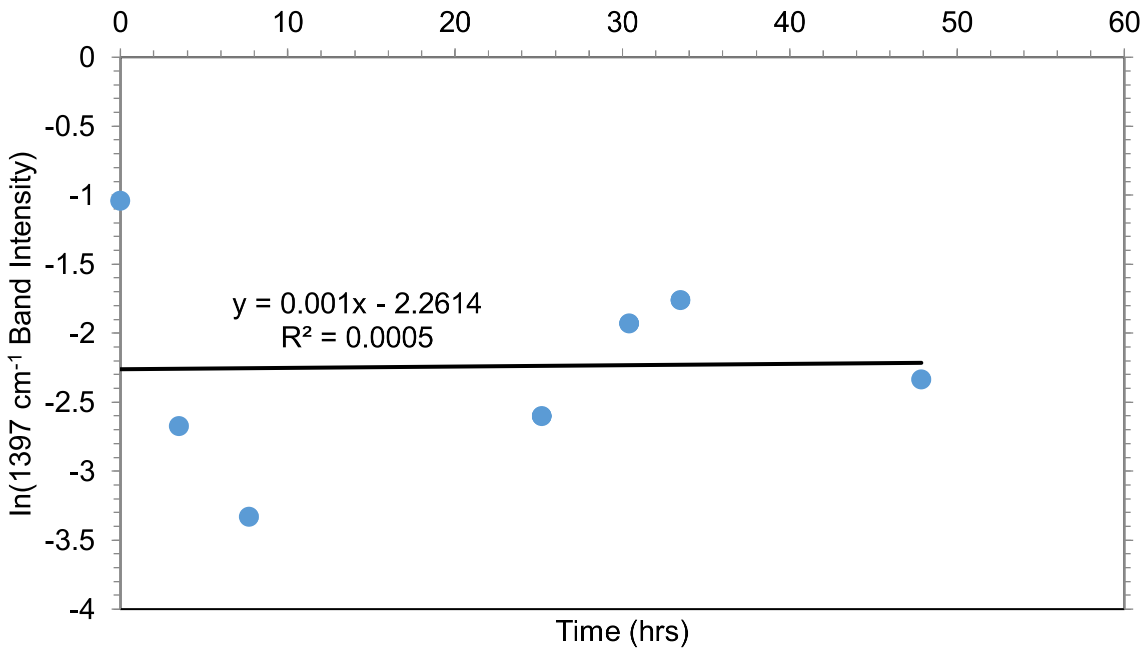

Figure S12. Natural logarithm of 1397 cm^{-1} band intensity versus time and linear regression for TS C.

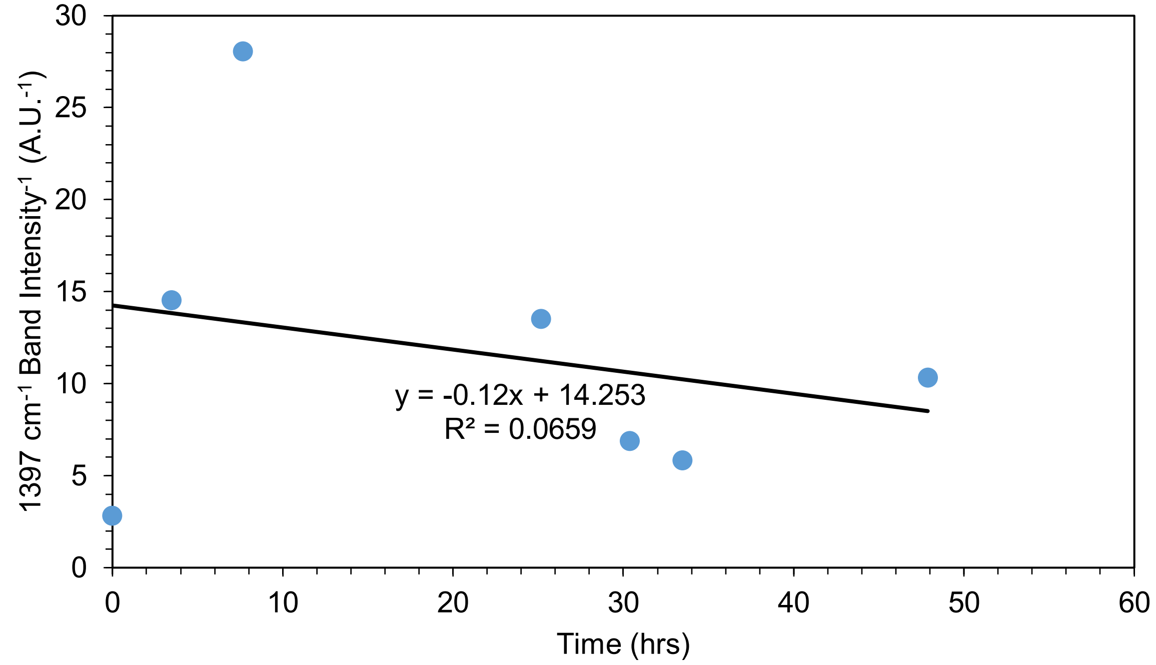

Figure S13. Inverse of 1397 cm^{-1} band intensity versus time and linear regression for TS C.

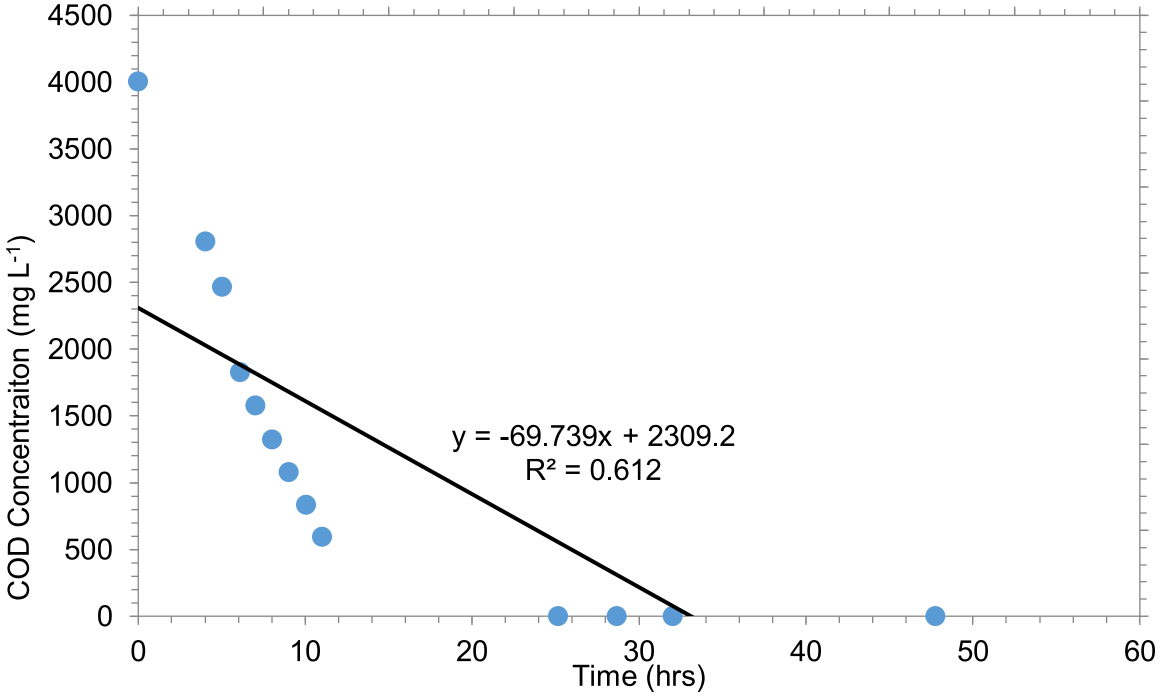

Figure S14. COD concentration vs time and linear regression for TS D.

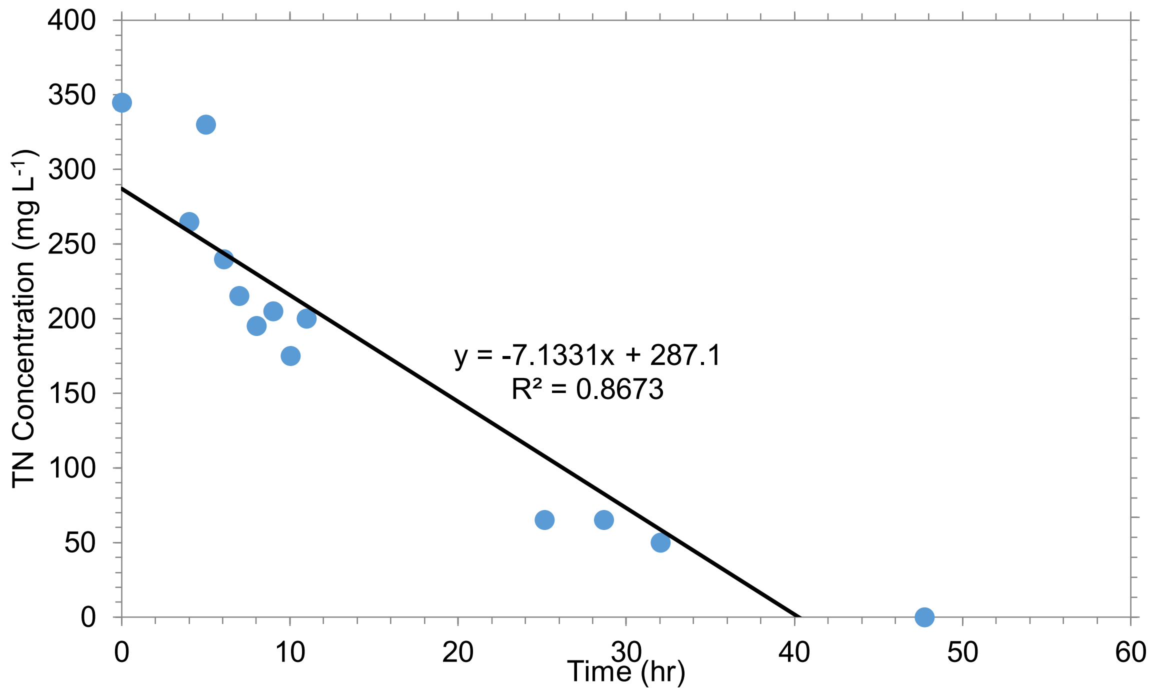

Figure S15. TN concentration vs time and linear regression for TS D.

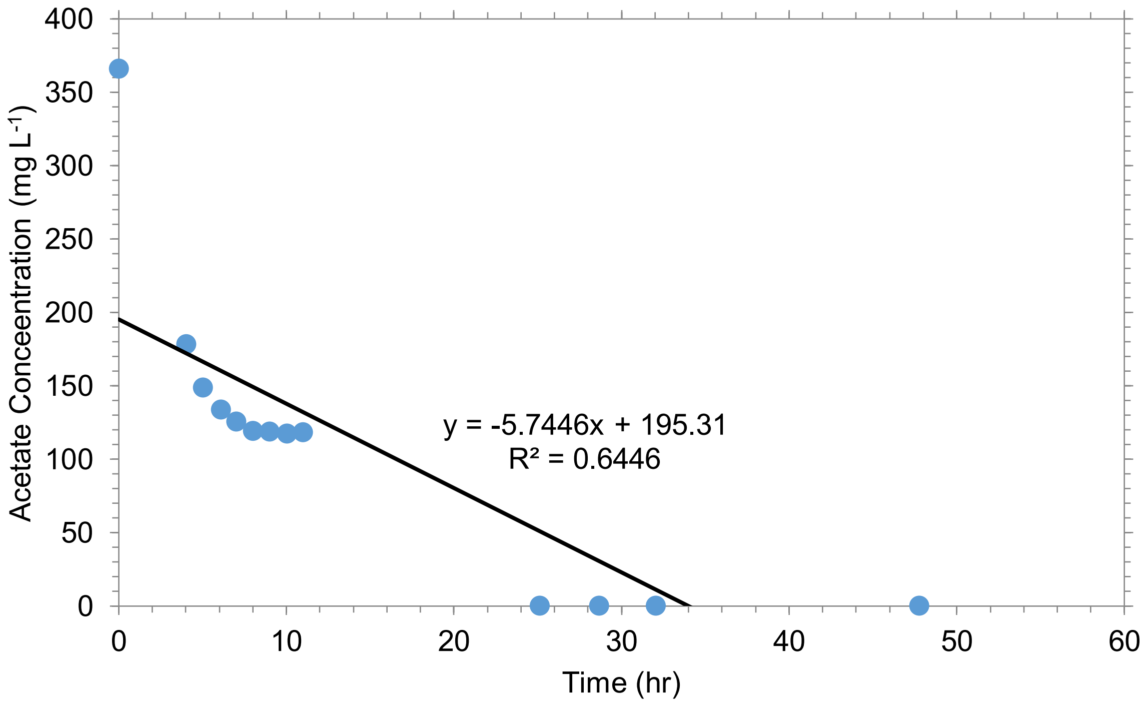

Figure S16. Acetate concentration versus time and linear regression for TS D.

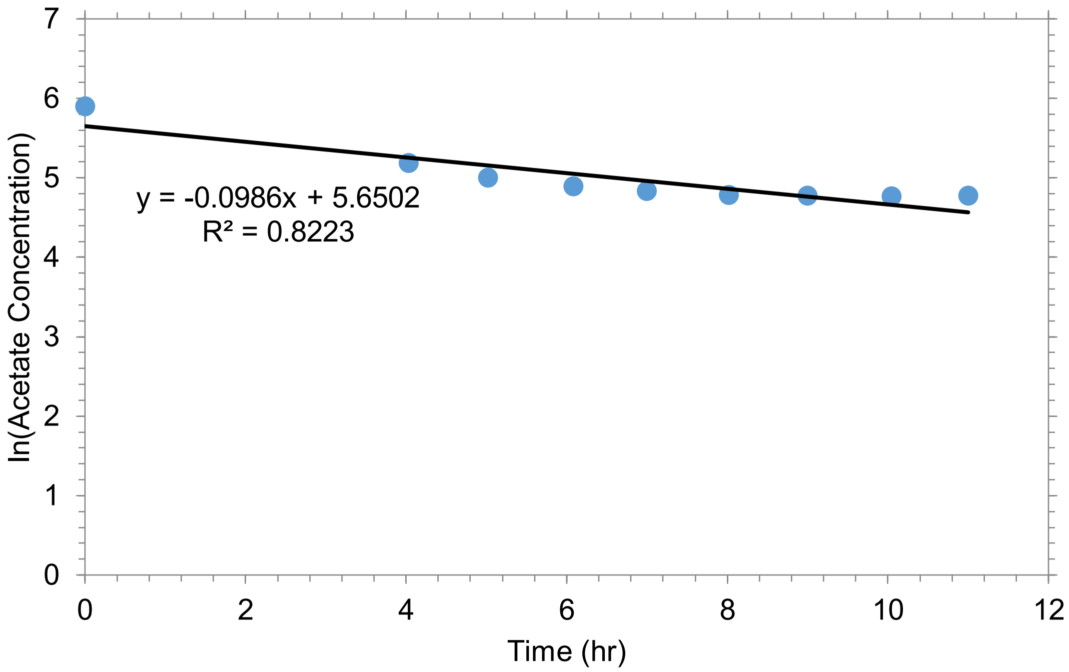

Figure S17. Natural logarithm of acetate concentration versus time and linear regression for TS D.

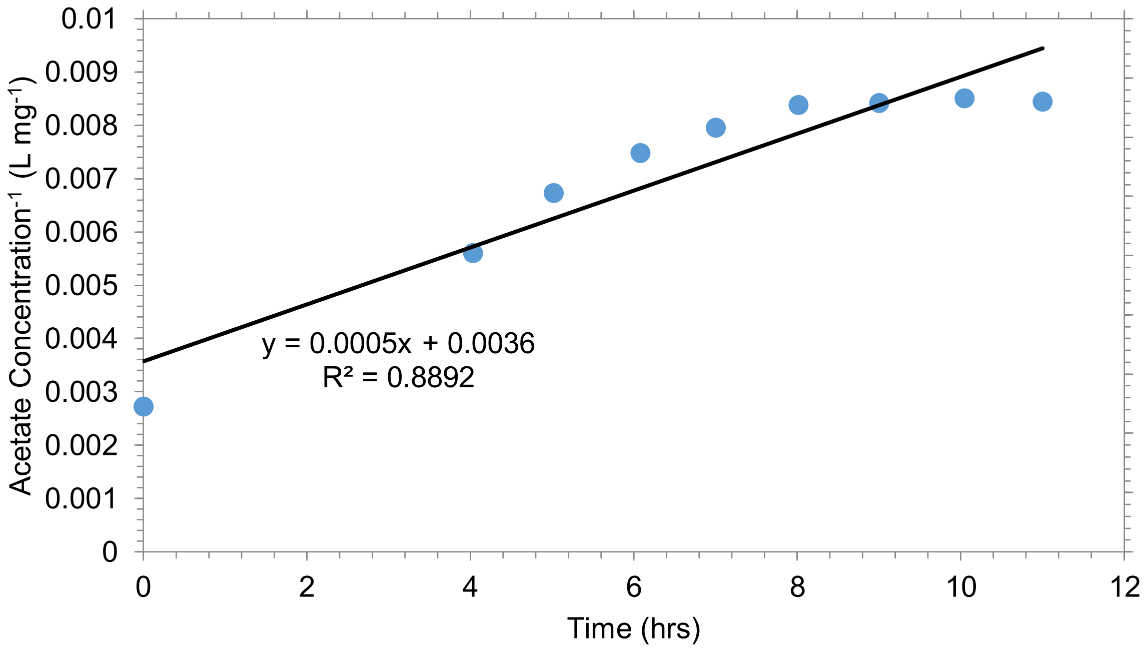

Figure S18. Inverse of acetate concentration and linear regression for TS D.

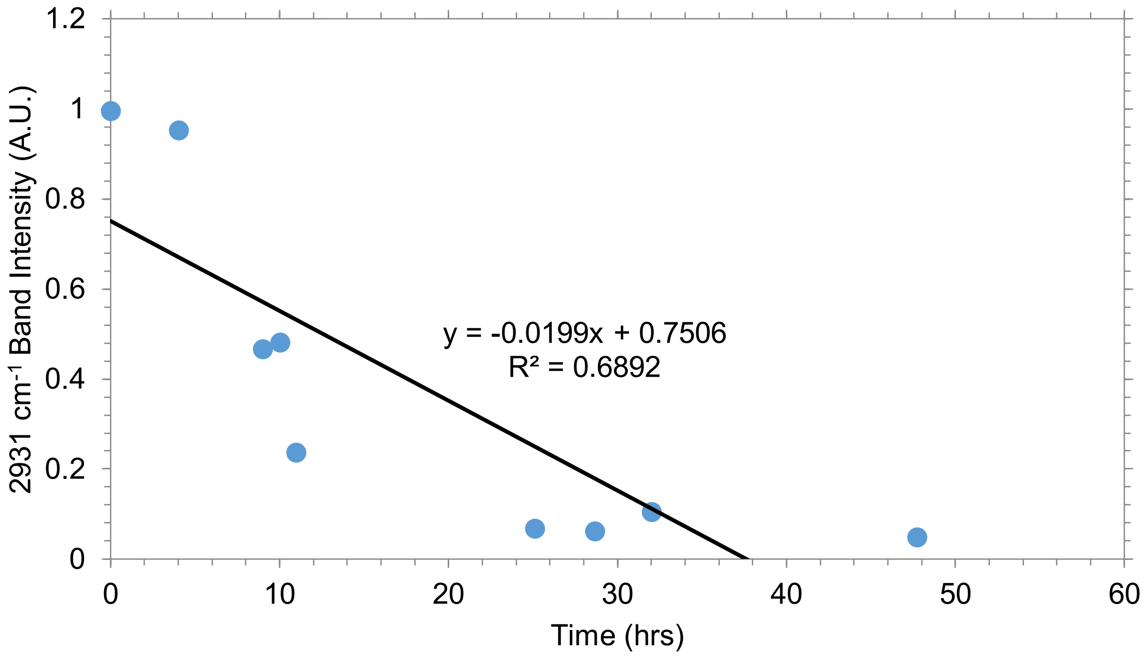

Figure S19. 2931 cm^{-1} band intensity versus time and linear regression for TS D.

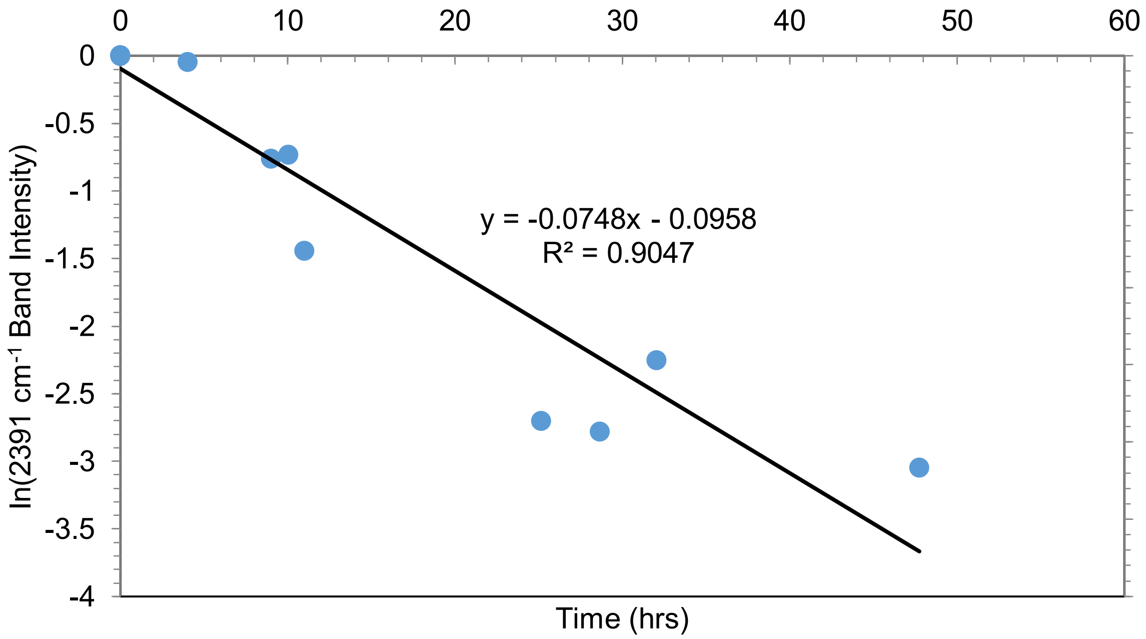

Figure S20. Natural logarithm of 2931 cm^{-1} band intensity versus time and linear regression for TS D.

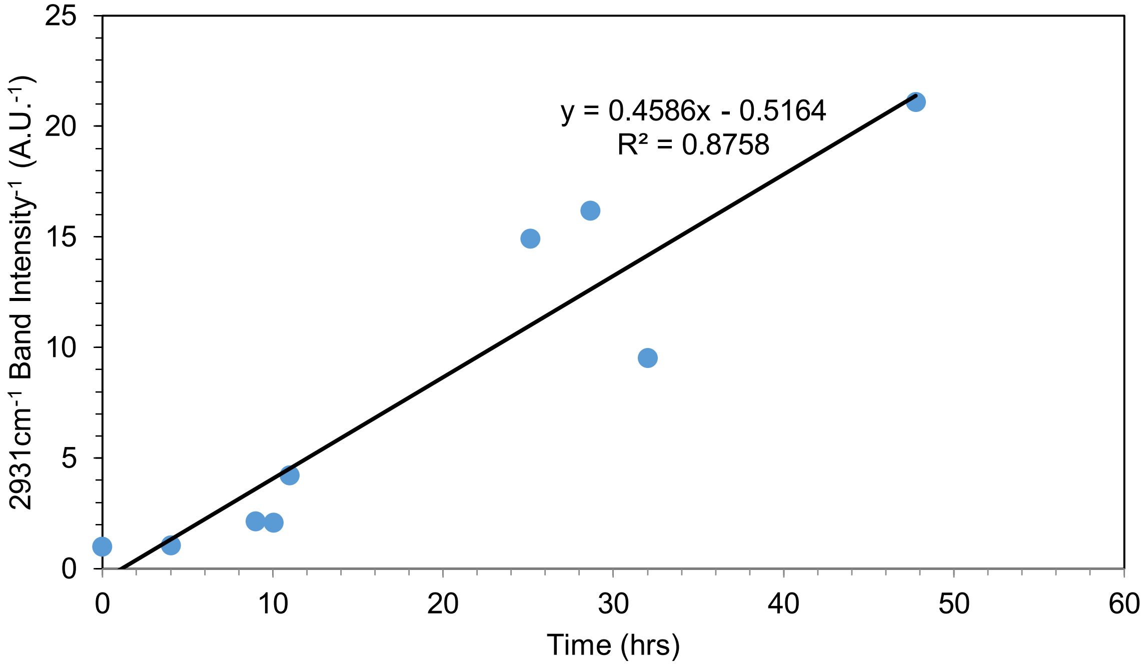

Figure S21. Inverse of 2931 cm^{-1} band intensity versus time and linear regression for TS D.

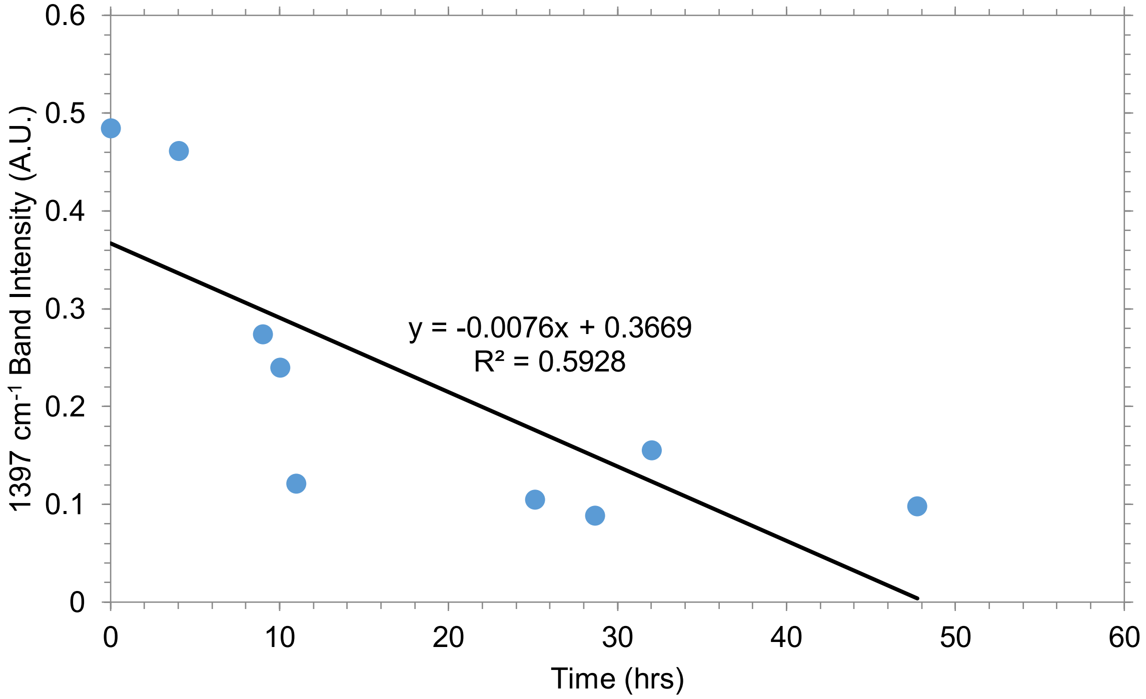

Figure S22. 1397 cm^{-1} band intensity versus time and linear regression for TS D.

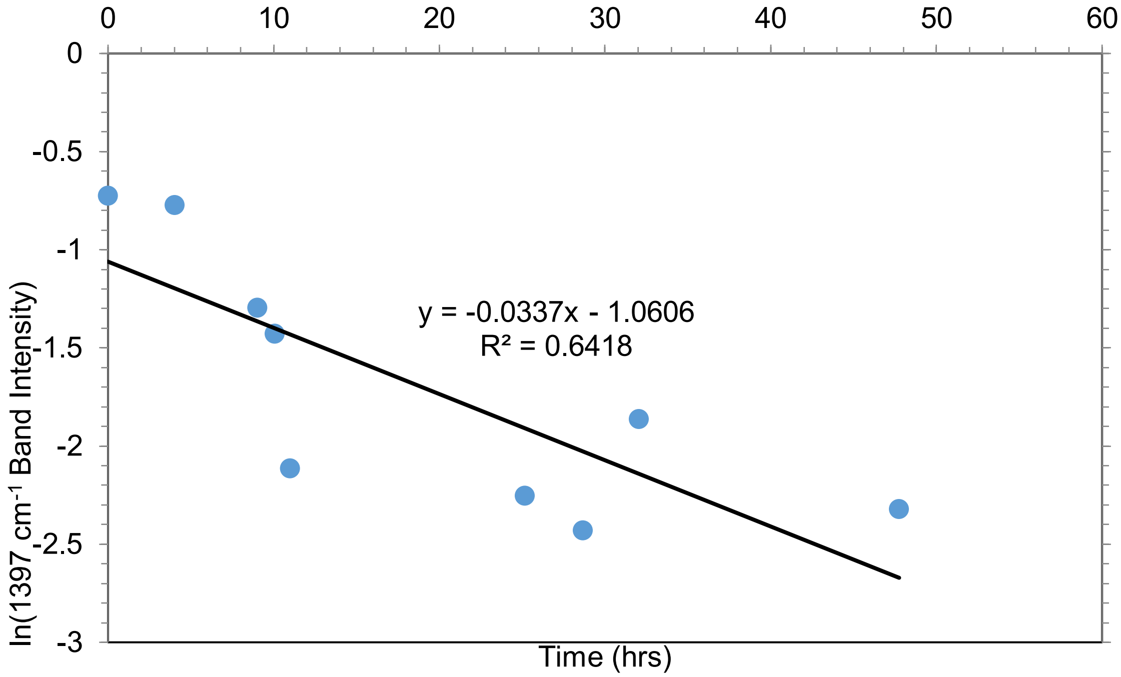

Figure S23. Natural logarithm of 1397 cm^{-1} band intensity versus time and linear regression for TS D.

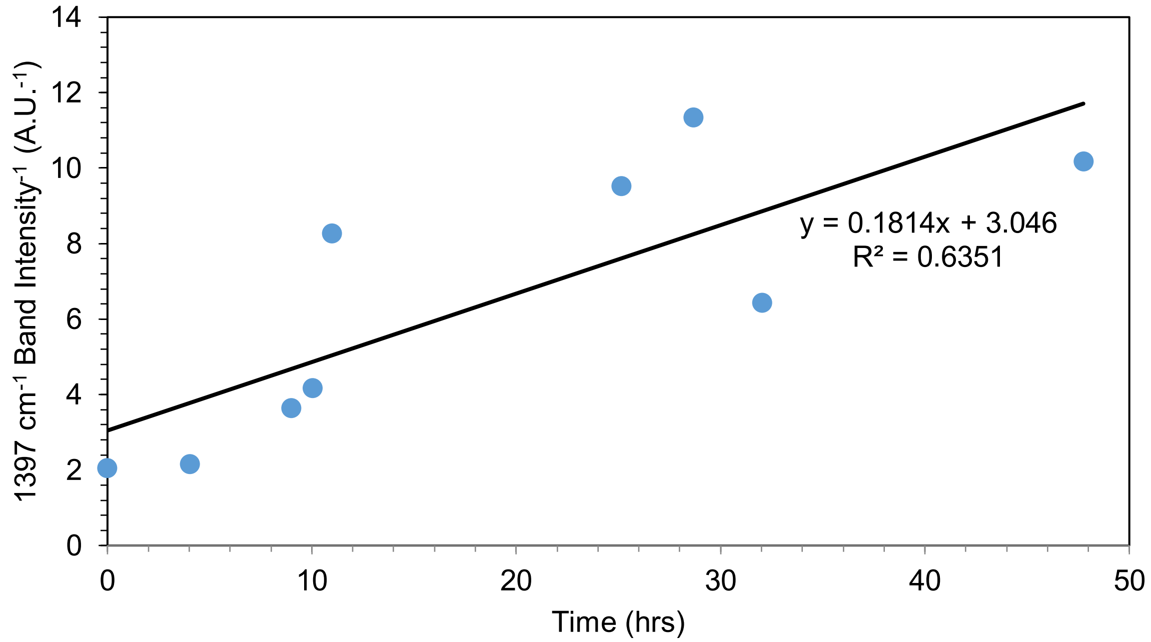

Figure S24. Inverse of 1397 cm^{-1} band intensity versus time and linear regression for TS D.

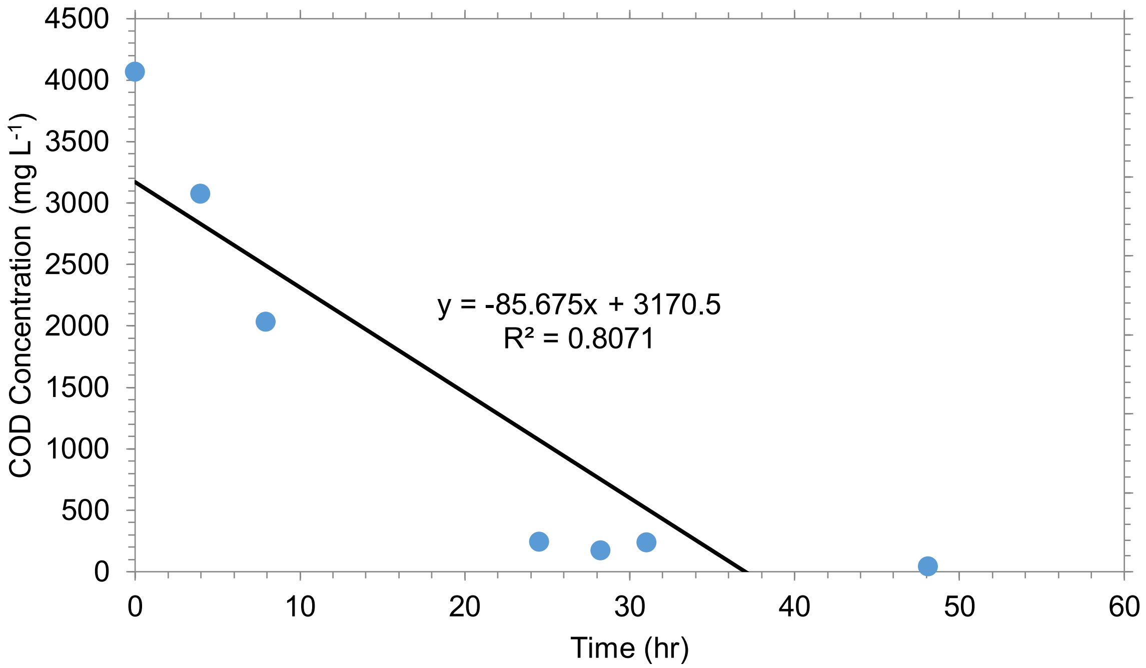

Figure S25. COD concentration vs time and linear regression for TS E.

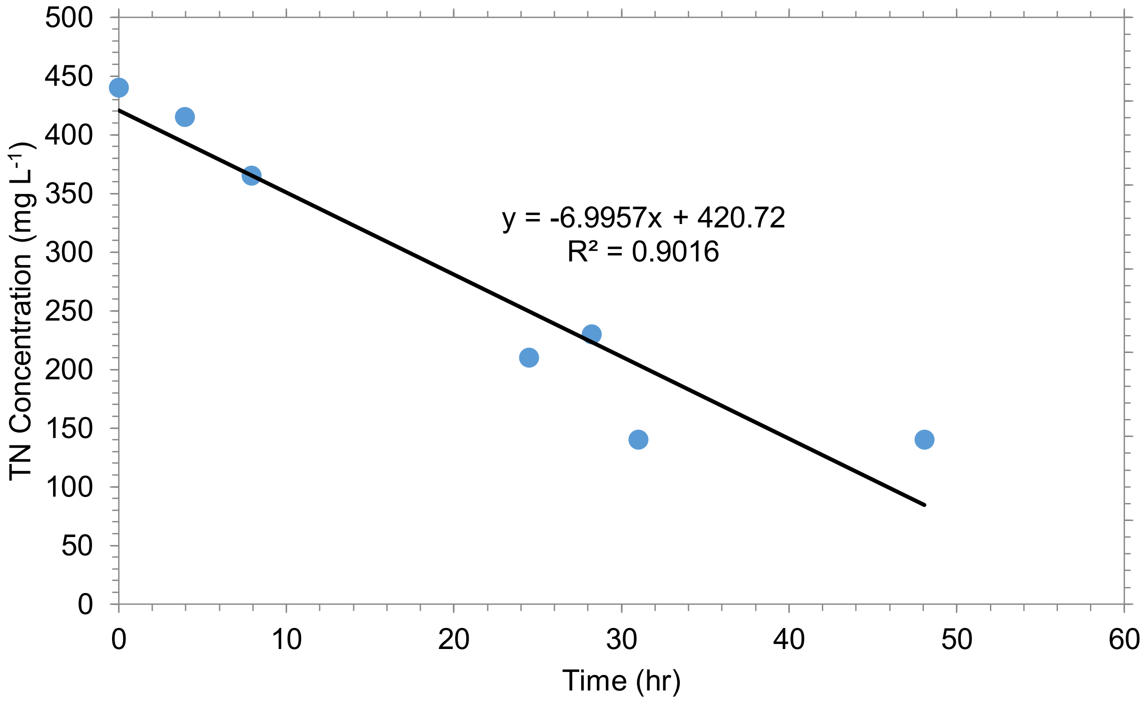

Figure S26. TN concentration vs time and linear regression for TS E.

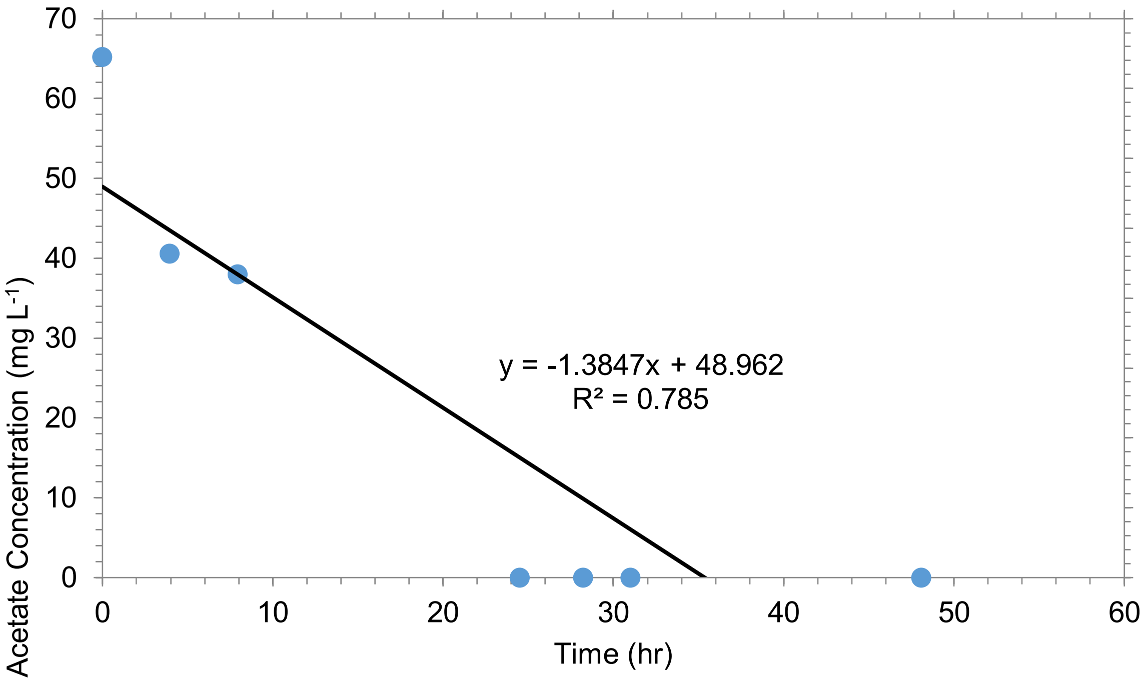

Figure S27. Acetate concentration versus time and linear regression for TS E.

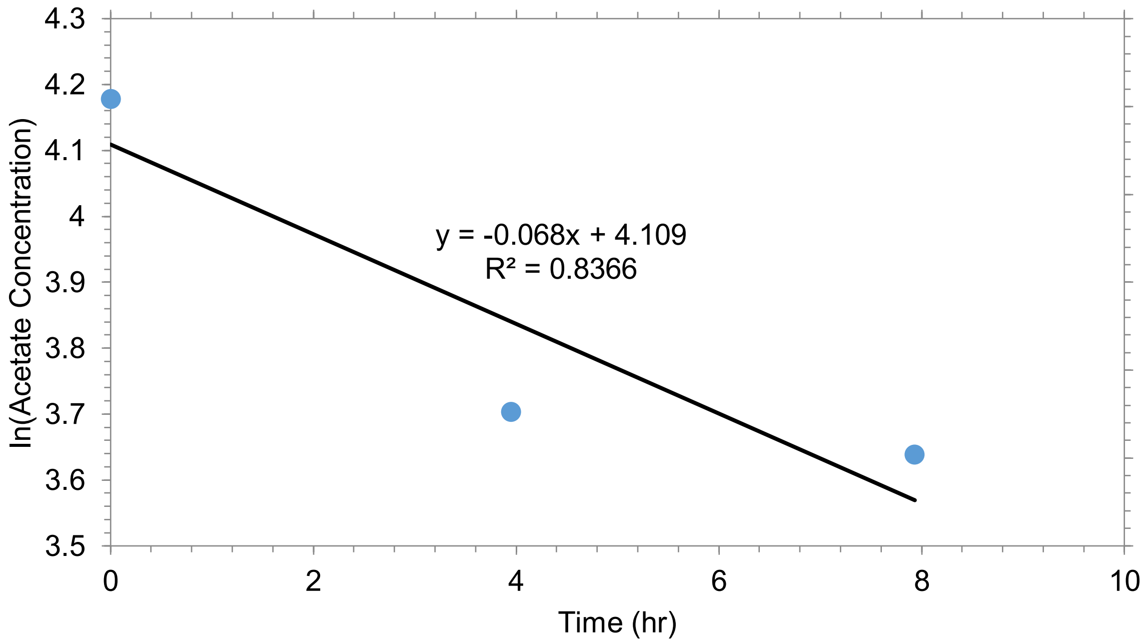

Fig S28. Natural logarithm of acetate concentration versus time and linear regression for TS E.

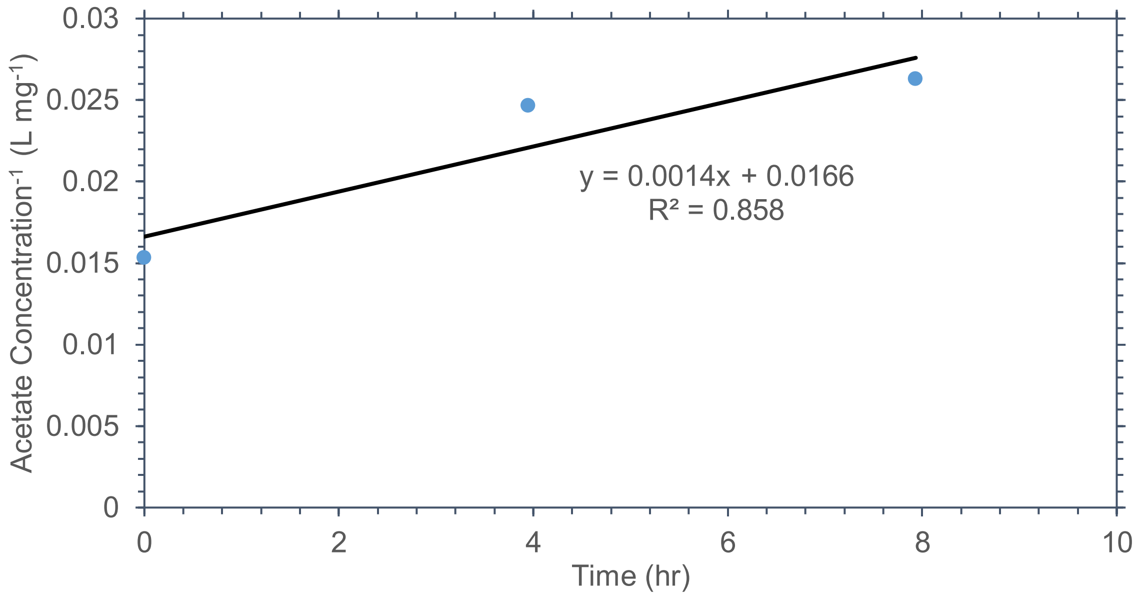

Figure S29. Inverse of acetate concentration and linear regression for TS E.

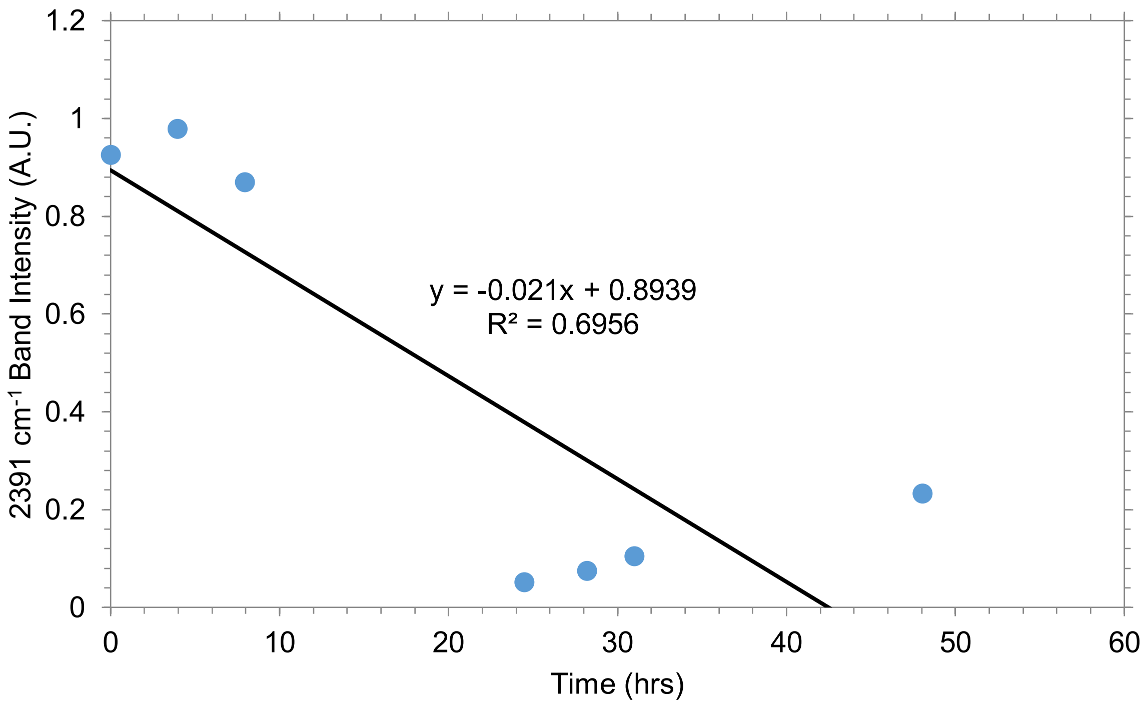

Figure S30. 2931 cm^{-1} band intensity versus time and linear regression for TS E.

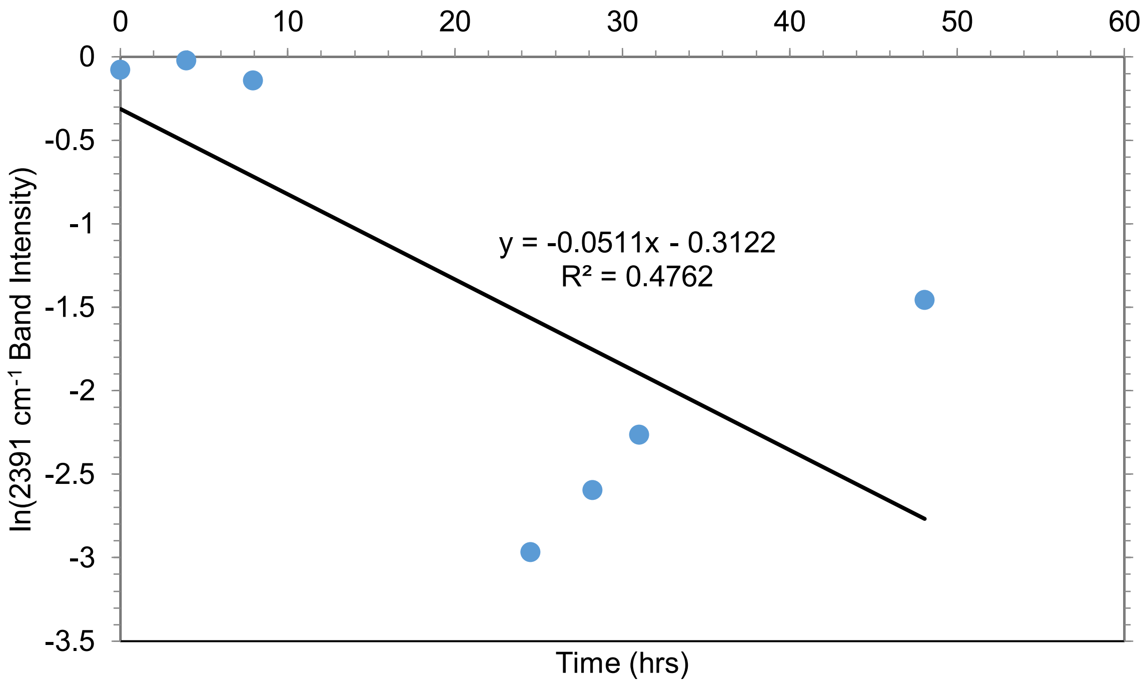

Figure S31. Natural logarithm of 2931 cm^{-1} band intensity versus time and linear regression for TS E.

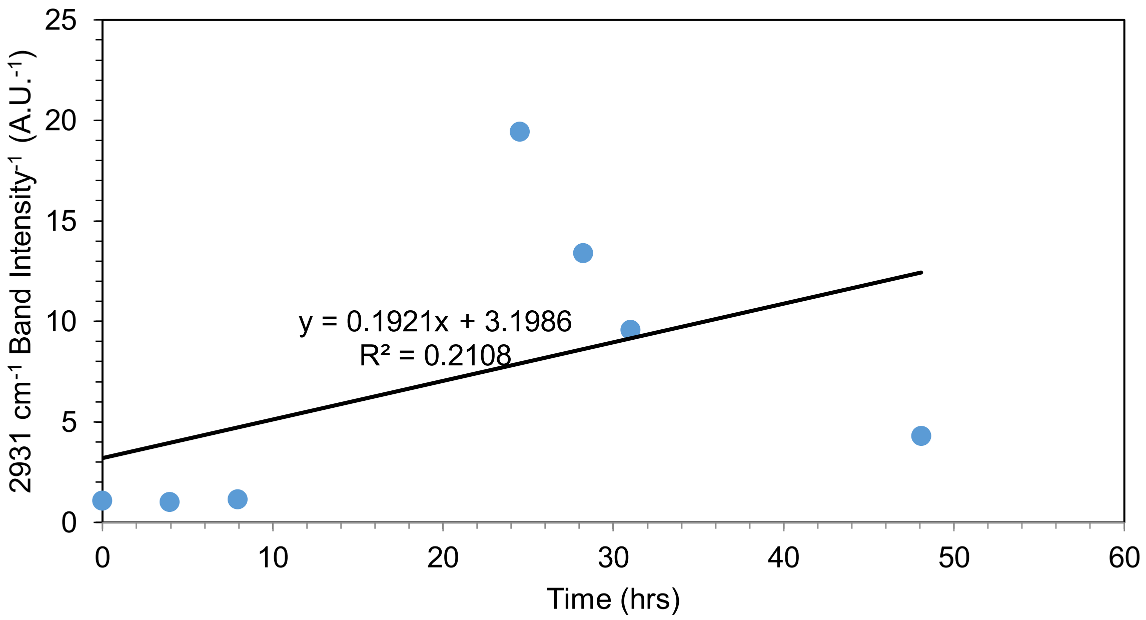

Figure S32. Inverse of 2931 cm^{-1} band intensity versus time and linear regression for TS E.

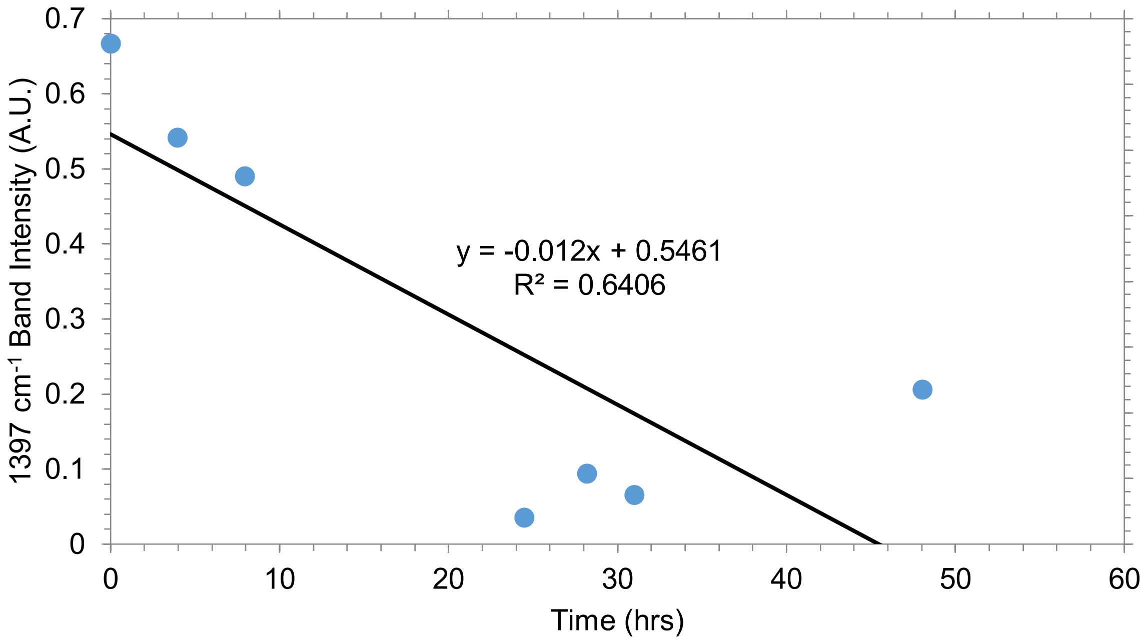

Figure S33. 1397 cm^{-1} band intensity versus time and linear regression for TS E.

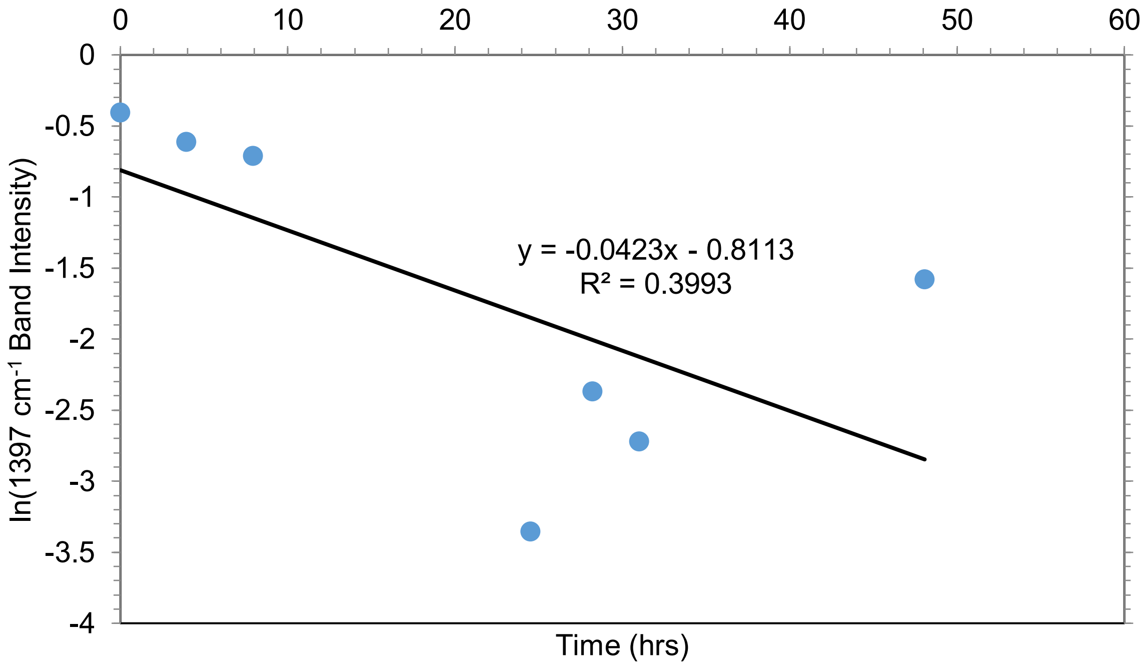

Figure S34. Natural logarithm of 1397 cm^{-1} band intensity versus time and linear regression for TS E.

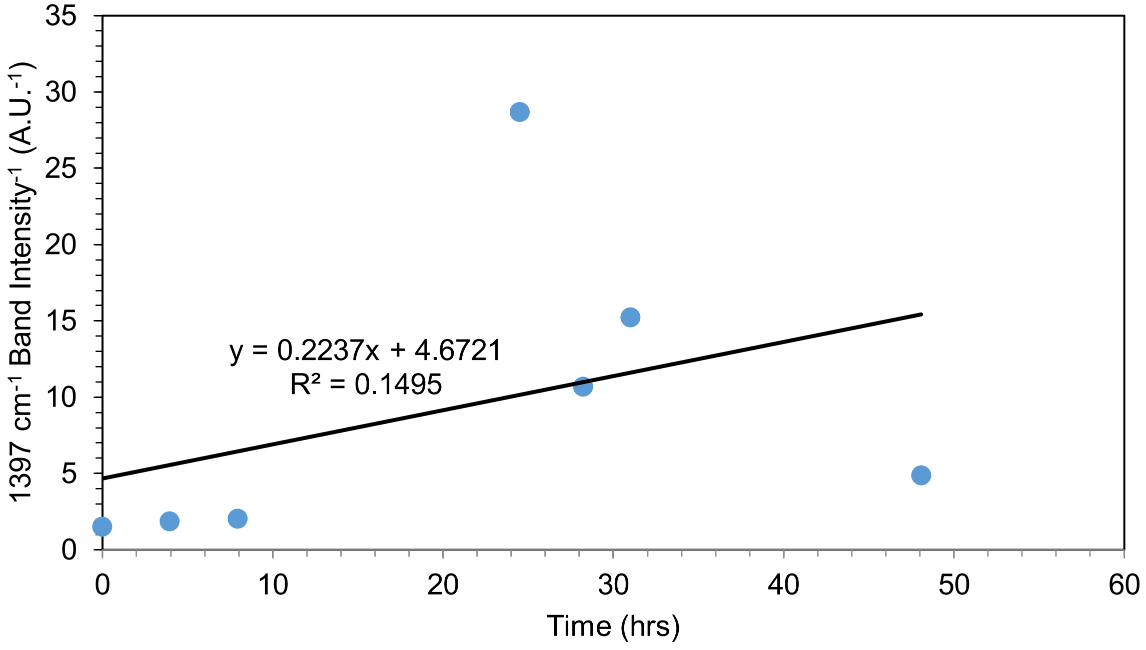

Figure S35. Inverse of 1397 cm^{-1} band intensity versus time and linear regression for TS E.

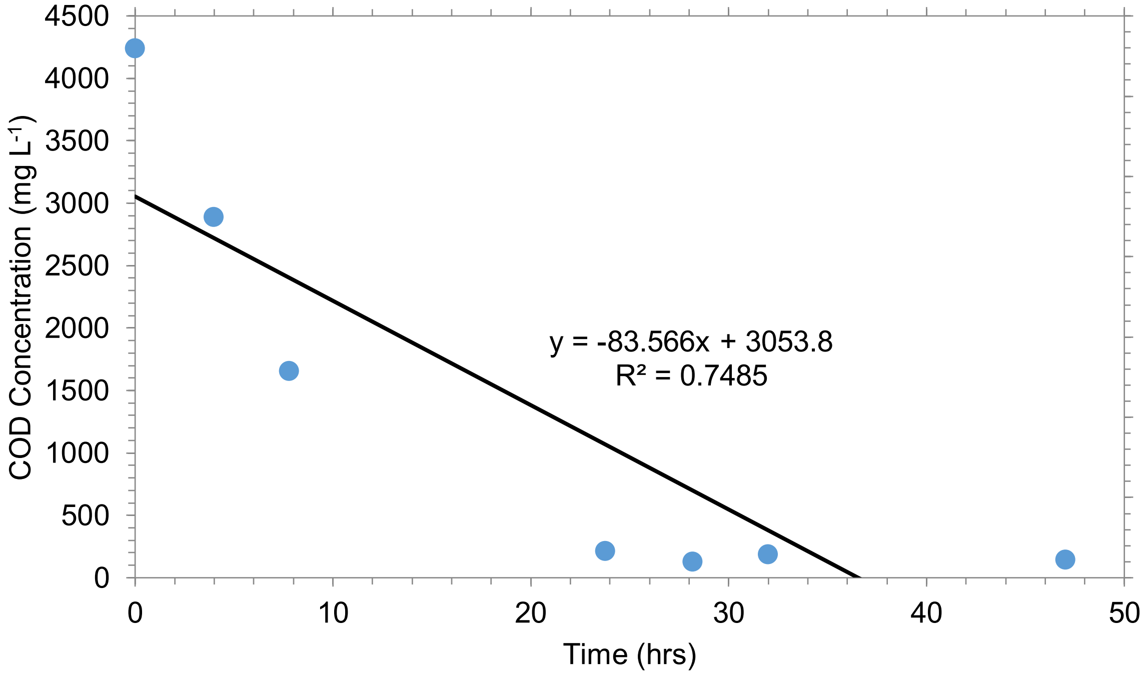

Figure S36. COD concentration vs time and linear regression for TS G.

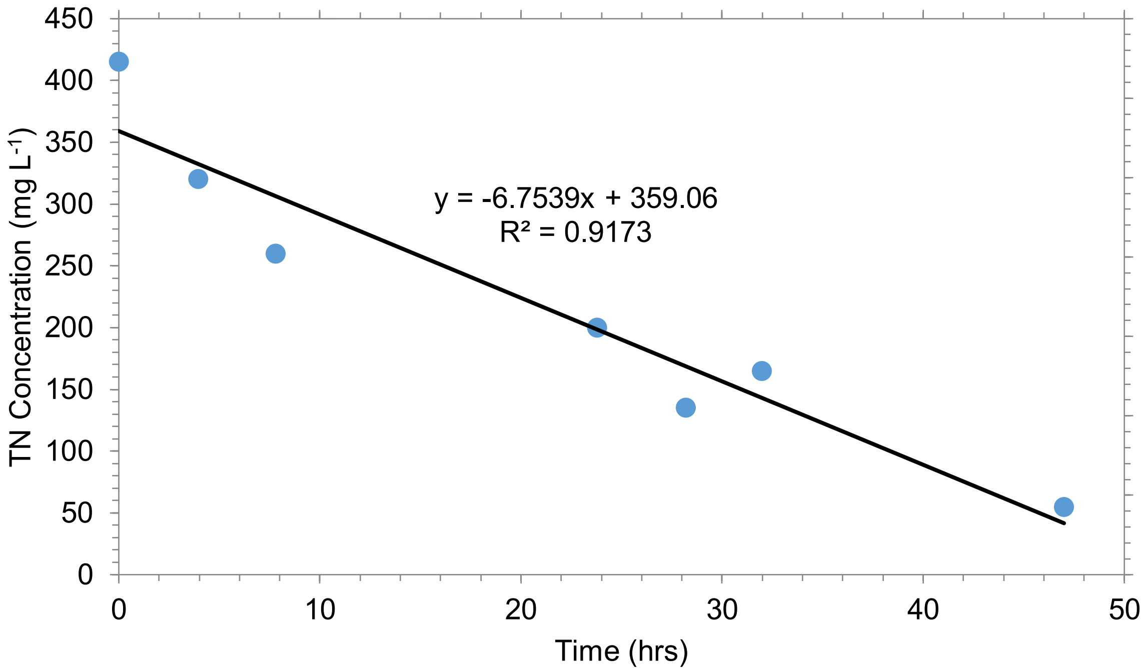

Figure S37. TN concentration vs time and linear regression for TS G.

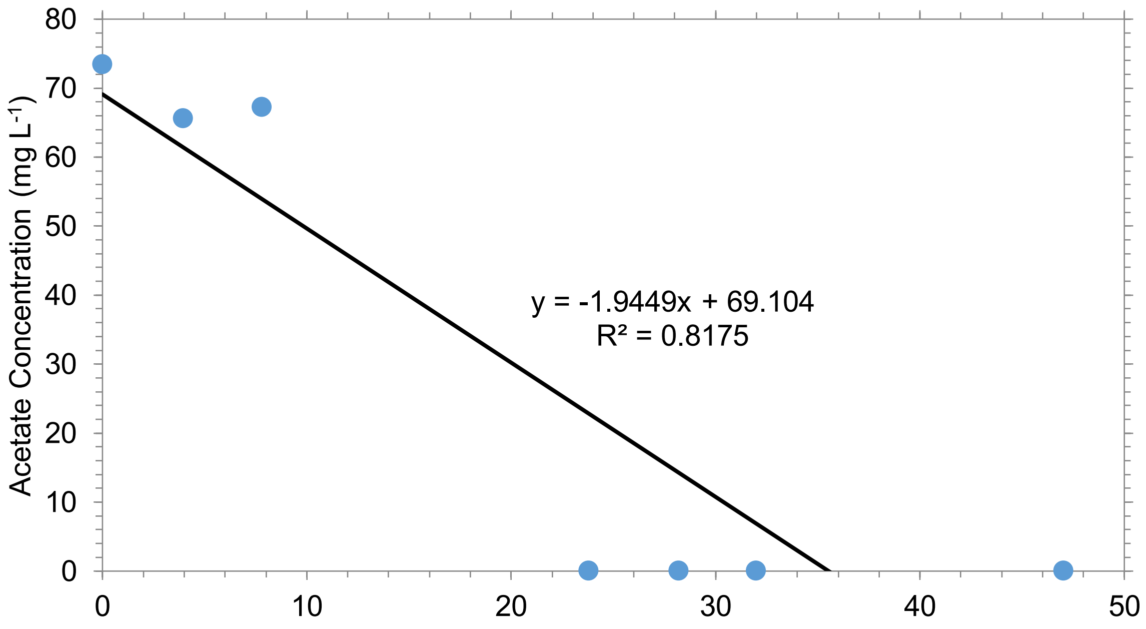

Figure S38. Acetate concentration versus time and linear regression for TS G.

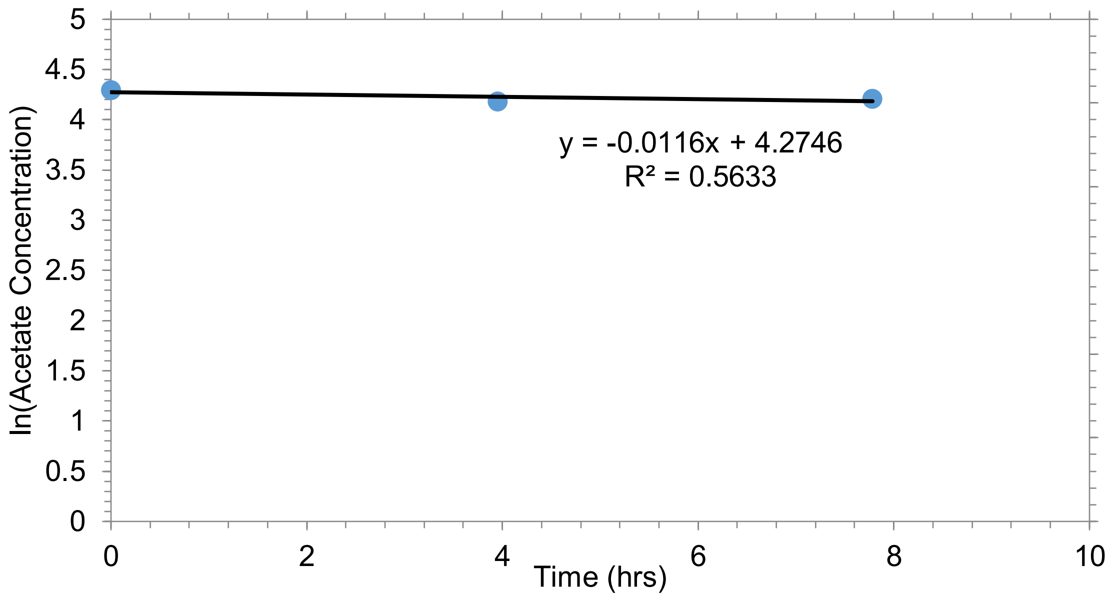

Figure S39. Natural logarithm of acetate concentration versus time and linear regression for TS G.

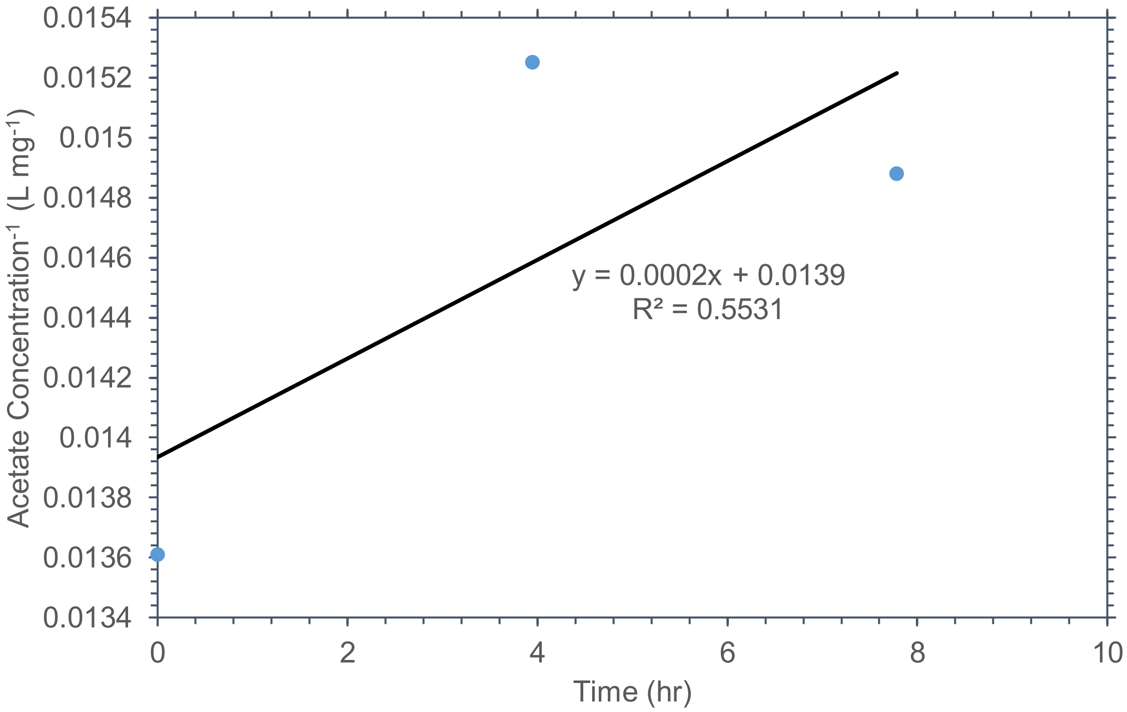

Figure S40. Inverse of acetate concentration and linear regression for TS G.

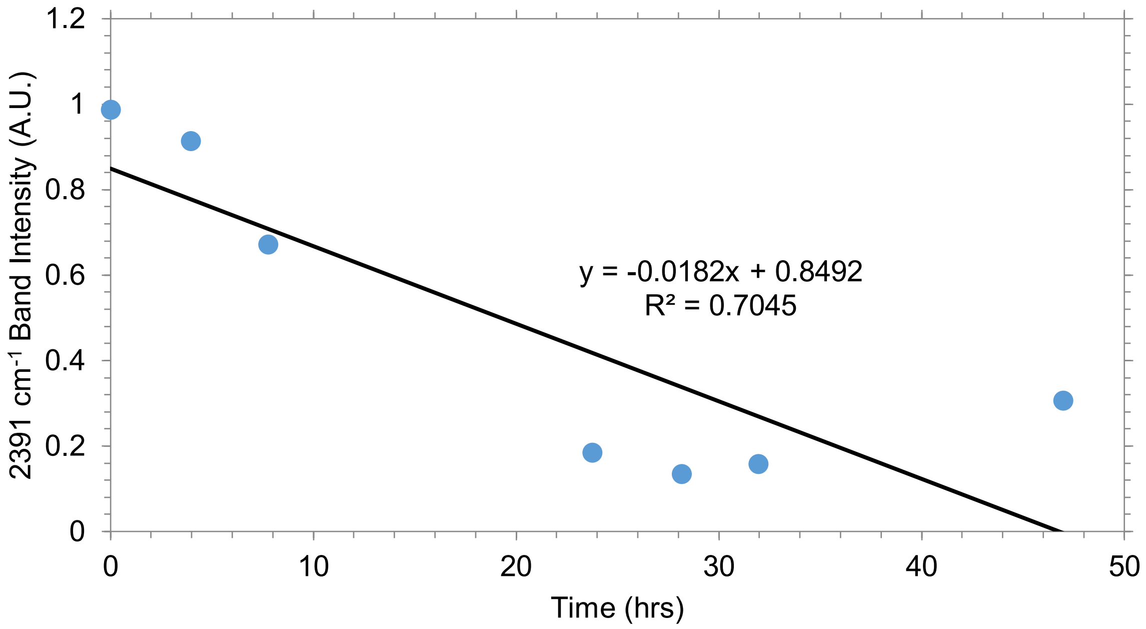

Figure S41. 2931 cm^{-1} band intensity versus time and linear regression for TS G.

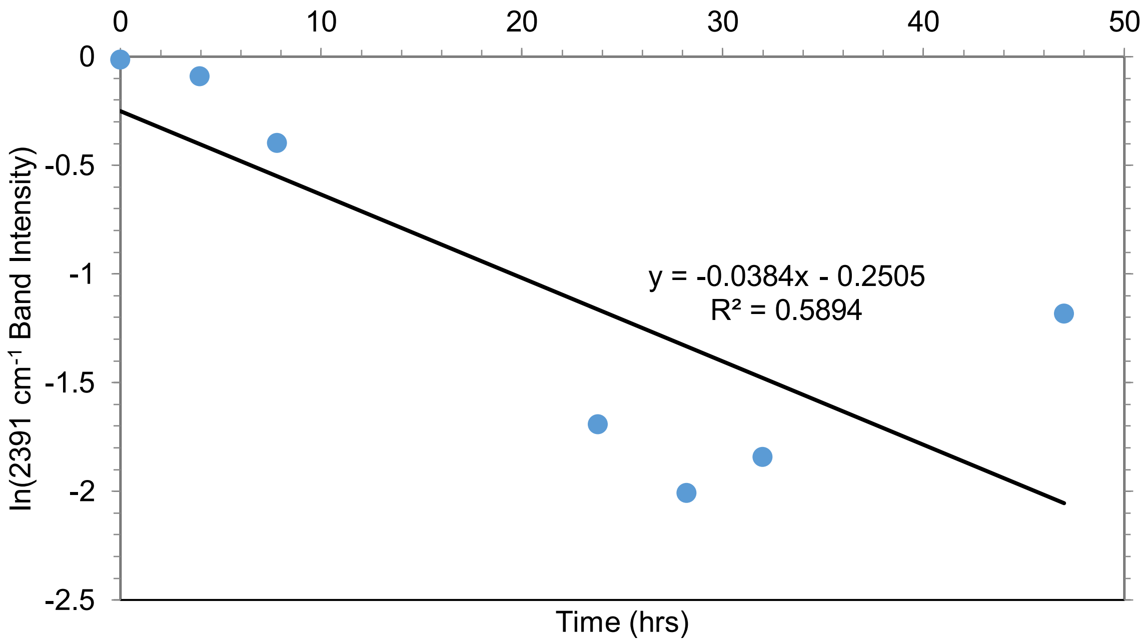

Figure S42. Natural logarithm of 2931 cm^{-1} band intensity versus time and linear regression for TS G

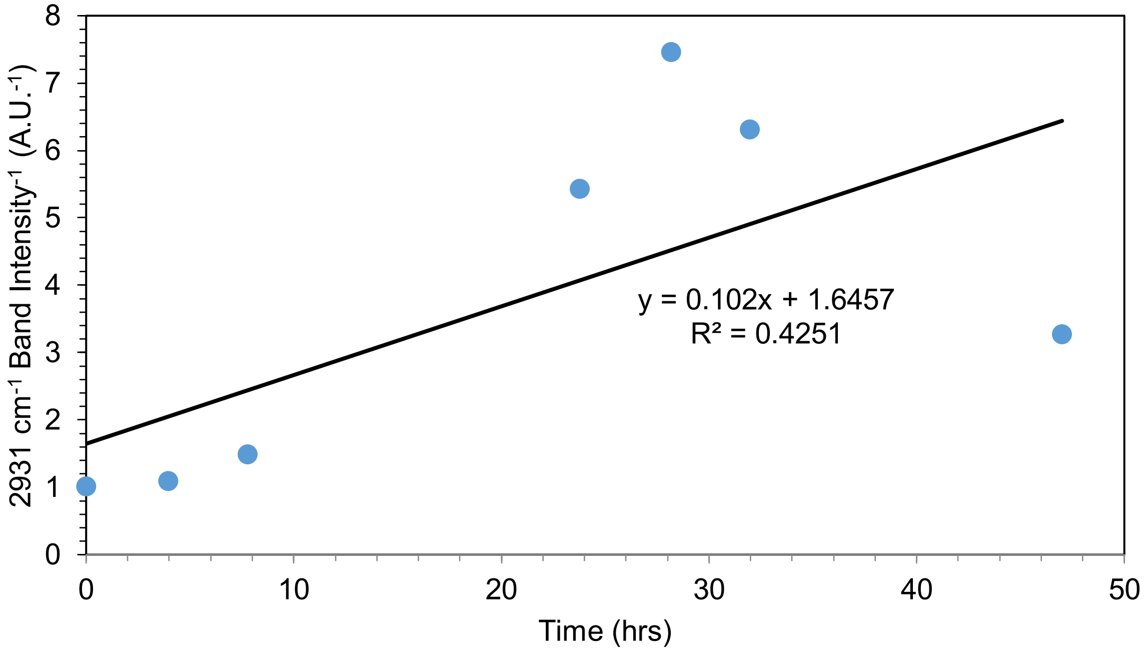

Figure S43. Inverse of 2931 cm^{-1} band intensity versus time and linear regression for TS G**.**

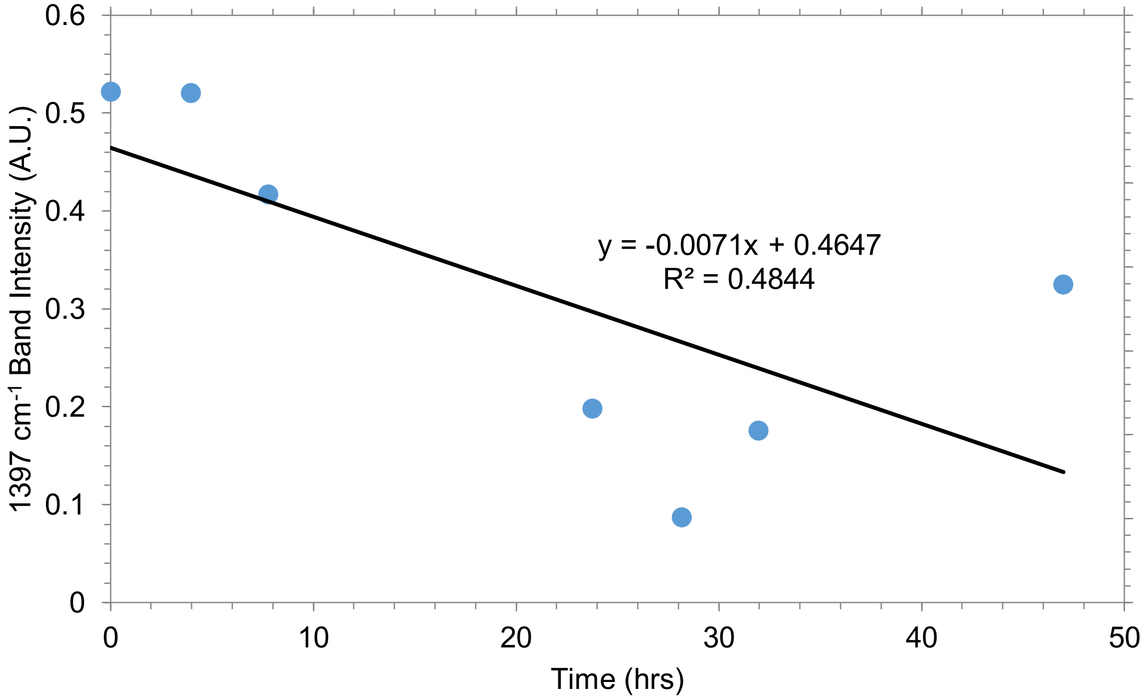

Figure S44. 1397 cm^{-1} band intensity versus time and linear regression for TS G.

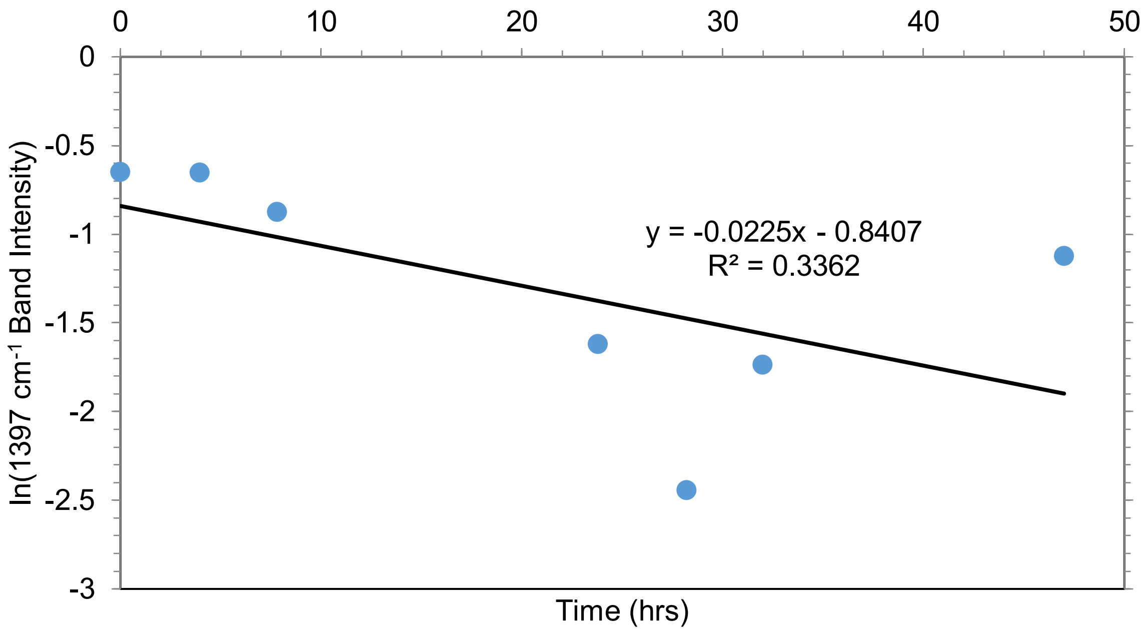

Figure S45. Natural logarithm of 1397 cm^{-1} band intensity versus time and linear regression for TS G.

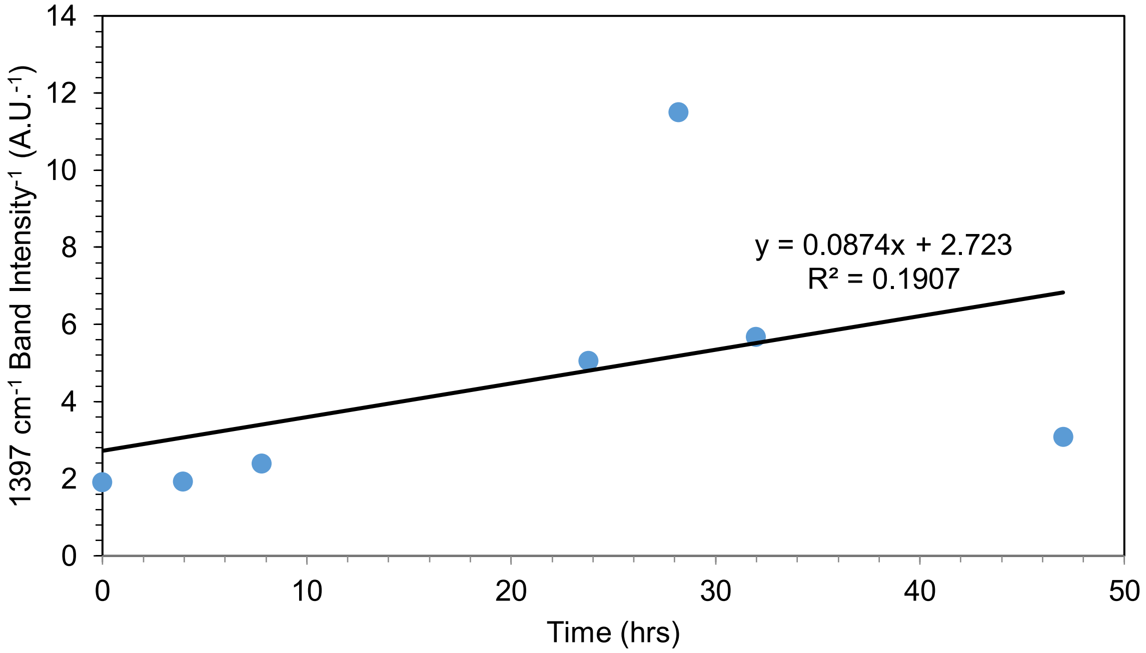

Figure S46. Inverse of 1397 cm^{-1} band intensity versus time and linear regression for TS G.
